# The effect of scene articulation on transparent layer constancy

**DOI:** 10.1167/jov.21.10.16

**Published:** 2021-09-22

**Authors:** Charlotte Falkenberg, Franz Faul

**Affiliations:** 1 Institut für Psychologie, Universität Kiel, Kiel, Germany

**Keywords:** transparency, transparent layer constancy, scene constancy, articulation, numerosity, color constancy

## Abstract

In this article, we examine the influence of scene articulation on transparent layer constancy. We argue that the term *articulation* may be understood as an aspect of the more general concept *naturalness of a stimulus* that relates to the degree of enrichment compared with a minimal stimulus and to the extent to which a stimulus contains regularities that are typically found in natural scenes. We conducted two matching experiments, in which we used strongly reduced scenes and operationalized articulation by the number of background reflectances (*numerosity*). The results of the first experiment show that higher numerosity actually leads to an increase in transparent layer constancy when reflectances are randomly drawn from a fixed population. However, this advantage disappears if the spatial mean and the variation of the subset colors are controlled as in our second experiment. Furthermore, our results suggest that the mechanism underlying transparent layer constancy leads to a rather stable compromise between two matching criteria, namely, proximal identity and constant filter properties according to our perceptual model. For filters with an additive component, which appear more or less hazy, we observed improved recovered filter properties and correspondingly higher degrees of transparent layer constancy, suggesting an additional mechanism in this type of filter.

## Introduction

If we look at transparent objects in everyday situations, for example, a colored drinking vessel, the perceptual properties that we attribute to such objects should ideally remain constant, regardless of changes in the viewing context. Such context changes may be caused by quantitative or qualitative changes in the illumination, but also by changes in the reflectance, the orientation, or simply the number of surfaces in the surround. It is well-known that, if an object moves against a variegated background, we usually do not perceive any changes in the objects’ properties (cf. Whittle, 2003). This kind of invariance is called *background-independent constancy* (e.g., Gilchrist et al., 1999) or *scene invariance* (e.g., Mausfeld & Andres, 2002) in differentiation to illumination-independent constancy or illumination invariance.

The question of which factors lead to more or less perceptual constancy has often been addressed in the past. In the domains of object lightness and object color, the term *articulation* was often used to describe some of the factors that lead to increased lightness or color constancy. Initially introduced by Katz and then adopted by the Gestalt psychologists, the term *articulation* (*Gesichtsfeldgliederung*, Katz, 1930) referred to scene complexity. Henneman (1935) wrote: “This field complexity factor has received many terms—‘Gesichtsfeldgliederung,’ articulation, stimulus constellation, and others.” Later, *articulation* was often equated with *numerosity*, that is, the number of reflectances or color patches in the stimulus (for a review see Gilchrist & Annan, 2002). However, the term has also been extended to other stimulus attributes that were found to increase lightness or color constancy (e.g., see Maloney & Schirillo, 2002).

Our aim in the present article is to investigate the role of articulation in transparent layer constancy. To this end, we first consider different theoretical perspectives on *articulation* and identify the variables on which they focus. Second, we summarize key results about how these variables affect the accuracy of illumination estimation. This question is related directly to our main topic, transparent layer constancy, which has been shown to also depend on perceived illumination color (Falkenberg & Faul, 2019a). Given the close similarity between color constancy and transparent layer constancy, it seems useful to examine the relevant literature on color constancy.

As has already been pointed out in the literature, *articulation* is somewhat difficult to capture and the lack of a consistent definition was criticized (e.g., Henneman, 1935; Maloney & Schirillo, 2002). Although certain stimulus attributes have repeatedly been found to increase the degree of constancy, no clear taxonomy of the relevant stimulus attributes has emerged and the underlying mechanisms remain largely unknown. In some cases, even contradictory results were found for individual stimulus attributes, necessitating the introduction of additional moderating variables.

A natural and plausible approach to clarify the term articulation seems to be to relate it to the complexity of a scene, because we seem to have an intuitive understanding of scene complexity and the degree of scene complexity can be regarded as a continuum between a minimal stimulus and typical natural scenes. However, the difficulty with this approach is that the degree of naturalness of a stimulus can manifest itself in very different dimensions and at different levels, which cannot easily be mapped into a straightforward taxonomy (cf., Radonjić, Cottaris, & Brainard, 2015a). Historically, the concept naturalness of a stimulus has been approached from two fundamentally different perspectives, which may be called the *stimulus-centered perspective* and the more holistic *scene-centered perspective*.

From the *stimulus-centered perspective*, scene complexity can be understood as the degree of enrichment of a minimal stimulus. Early studies focused on the color appearance of isolated light patches in complete darkness and this strategy allowed the investigation of basal functions and capabilities of brightness and color vision. However, it was soon noticed that color appearance is not a simple function of the physical input, but in general changes drastically as soon as a second light stimulus is added. To determine the mechanisms underlying context related changes of color appearance, the stimulus complexity was stepwise increased from simple center-surround stimuli to various types of color mosaics. In this approach, the focus is on the color distribution in local neighborhoods and considers, for instance, the number of differently colored patches, local color contrasts, or simple moments of the color distribution like the mean or the variance. By increasing the number or the variance of colors in the stimulus, the stimulus tends to approximate more closely the color distribution of a natural environment, in which many different objects with varying surface properties occur. In this context, naturalness can be understood in terms of the statistics of the chromatic properties of the stimulus. Correspondingly, this approach often focuses on low-level processes and mechanisms.

The *scene-centered perspective*, in contrast, emphasizes the scene interpretation in terms of relevant internal categories of the visual system. In this approach, the focus is on the meaningful interpretation of the visual input, for example, with regard to the prevailing illumination, three-dimensional (3D) surface orientation, and ultimately the shape and identity of objects. The problem of color constancy, that is, how a stable perception of object colors is possible despite changes in the scene context, originally arose from a more holistic perspective that separated the two internal concepts of object color and illumination color (e.g., Helmholtz, 1867; Katz, 1930). In this approach, it is implicitly or explicitly assumed that the basis for color constancy lies in a correct scene interpretation that includes an internal estimate of illumination color. To enable a successful interaction with objects in the environment, the available input, in which object reflectance and illumination are inseparably confounded, must be interpreted in a way that the results correspond, at least functionally, to the true properties of objects in the outside world.

However, this does not necessarily mean that scene-centered approaches use more complex scenes in investigations on color constancy. It has been suggested that even center-surround stimuli may trigger both representations, that is, object color and illumination color, in a rudimentary form (Bergström, 1977; Mausfeld & Niederée, 1993; Mausfeld, 1998; Walraven, 1976; Whittle, 1994). Thus, both approaches can finally result in the employment of identical stimuli, but the underlying theoretical considerations and the associated research questions may differ substantially.

### Effects of articulation on color constancy

In our brief review of previous work, we primarily consider the large number of studies that investigated the effect of different degrees of articulation or stimulus naturalness on *lightness* and *color constancy*, but *w*e also include some studies that focused on the effects of stimulus configuration on local *brightness* and *color appearance*. Because in transparent layer constancy the chromaticity of the illumination is of central importance while lightness anchoring plays a minor role, we mainly focus on color constancy.

The attributes influencing the naturalness of scenes can roughly be categorized as either colorimetric or figural. With regard to colorimetric attributes, previous studies frequently explored statistical properties of the stimuli, whereas studies on figural organization usually considered more complex aspects of scene interpretation.

#### Statistical distributions of stimulus chromaticities

Many studies have shown that an increase in numerosity, that is, an increase in the number of different surface reflectances or colors in the stimulus, leads to improved lightness or color constancy (e.g., Arend & Goldstein, 1987; Arend, Reeves, Schirillo, & Goldstein, 1991; Burzlaff, 1931; Gilchrist et al., 1999; Henneman, 1935; Katz, 1930; Linnell & Foster, 2002; Schirillo, 1999). However, there are also investigations that did *not* find an effect of numerosity on the degree of constancy (e.g., Amano, Foster, & Nascimento, 2005; Arend & Reeves, 1986; Radonjić, Cottaris, & Brainard, 2015b; Wedge-Roberts et al., 2020) or came to ambiguous results (e.g., Kraft, Maloney, & Brainard, 2002; Radonjić & Gilchrist, 2013).

The data on the effectiveness of numerosity are thus somewhat inconclusive, which may partly reflect the fact that very different stimulus materials were used. Numerosity was examined with both homogeneous color patches on the monitor as well as with physical papers and illuminations. However, the difficulties in drawing definitive conclusions about the effectiveness of numerosity cannot be attributed solely to such variations in the degree of naturalness of the stimuli, because inconsistent observations also occurred with virtually identical stimulus conditions, for example, with simple two-dimensional (2D) color patterns of homogeneous color patches.

Amano et al. (2005) found comparably high degrees of color constancy in an asymmetric matching task for stimuli containing either two or 49 patches (constancy index [CI] = 0.73 and *CI* = 0.72, respectively). Because the geometry and reflectances of the stimuli were identical under standard and test illuminations, the cone excitation ratios of adjacent patches were also more or less the same (cf., Foster & Nascimento, 1994), which is why the authors propose that the high degrees of constancy observed even for bipartite stimuli might be explained by relational color matching. However, if color constancy could be explained exclusively by identical cone excitation ratios, numerosity should have no influence at all, which obviously contradicts other findings which observed improved degrees of constancy for increased numerosity in comparable 2D stimuli (e.g., Arend & Goldstein, 1987; Arend, Reeves, Schirillo, & Goldstein, 1991; Gilchrist et al., 1999; Schirillo, 1999). The results of Rinner and Gegenfurtner (2002) obtained with well-articulated Mondrian patterns also speak against this interpretation. They found that arbitrarily exchanging the reflectance of the patches has no effect as long as the spatial average (and the variance) of the chromaticities remained the same. These observations indicate that the exact cone ratios at edges are not essential for color constancy under illumination changes.

To explain lightness and color constancy, it is often implicitly or explicitly assumed that an internal representation of the color and intensity of the illumination is derived from the input and somehow taken into account in the perception of the object's color or lightness. Accordingly, it has often been suggested that a positive effect of numerosity on color constancy can be attributed to the fact that it allows for improved illumination estimation.

Generally, the greater the number of randomly drawn reflectances in a scene, the better the average chromaticity of the reflected light approximates the color of the illumination. Andres (1997) showed that for an idealized situation in which all possible reflectances occur, the spectrum of the prevailing (homogenous) illumination can be reconstructed exactly from the resulting color codes. Of course, this situation is hypothetical; however, this result suggests that the color distribution in a scene can be used to infer properties of the illumination and that the accuracy of the estimate should increase with the number of reflectances that contribute to this distribution (Mausfeld & Andres, 2002). This assumption is in accordance with the findings of Linnell and Foster (2002) that the ability to detect an illumination change was improved when 49 instead of only nine color patches were drawn randomly from a fixed population (the Munsell Book of Color).

If the mean chromaticity were the only cue for the estimation of the illumination color, then numerosity should have no effect on the degree of constancy when the mean is kept constant. However, there is evidence that the estimation of the illumination color is not only determined from the chromatic average of the stimulus, but that specific higher order statistics, especially the chromatic variance, also have an influence. Jenness and Shevell (1995) found that the effect of color induction on the red–green equilibrium settings was smaller when the target patch was surrounded by a reddish background that contained sparse white and green dots than when it was presented against a homogenous reddish background with the same spatial average. Mausfeld and Andres (2002) systematically manipulated the degree of chromatic and luminance variance in inhomogenous surrounds and likewise observed that increasing the chromatic variation decreased induction significantly, whereas an isolated variation in luminance had no such effect. The results of both studies suggest that substantial chromatic variance restricts the range of possible illuminants to more neutral ones. In contrast, decreasing the chromatic variance increases the probability that a scene with a non-neutral spatial average will be interpreted as being illuminated by non-neutral light (Mausfeld, 1998). Golz and MacLeod (2002) report data suggesting that correlations between color codes could serve as additional cues for the illumination color (but see Granzier, Brenner, Cornelissen, & Smeets, 2005). These findings suggest that an increased numerosity in variegated scenes may reduce the ambiguity regarding the contributions of reflectance and illumination to the reflected light that is present in simple scenes, not only because of the larger number of independent random samples of reflectances, but also because higher order statistics of the color codes are available that further restrict the set of possible illuminations.

Another line of research examined how the local brightness or color appearance of a test patch depends on the color distribution in its surround. Here, it has often been observed that not only the spatial average, but also the chromatic variance has an influence (e.g., Adelson, 2000; Brenner & Cornelissen, 2002; Bressan & Actis-Grosso, 2006; Brown & MacLeod, 1997). Brown and MacLeod (1997), for instance, found that chromatic patches may appear much more saturated against an equiluminant, uniform gray surround than against a variegated surround with identical space-averaged color (gamut expansion effect). A related result was reported by Brenner and Cornelissen (2002) who found less color induction, that is, more frequent grey ratings, in a condition with color modulation in the surround. However, the results of Faul, Ekroll, and Wendt (2008) challenge an interpretation focusing solely on variance (and suggest a contribution of color scission) because they show that the gamut expansion effect can also be silenced without chromatic variation in the surround. In the achromatic domain, it was found that the simultaneous lightness contrast of a patch in a homogeneous surround is enhanced when the homogenous surround is replaced with an articulated one (Adelson, 2000; Bressan & Actis-Grosso, 2006; Gilchrist et al., 1999). Thus, it is evident that a chromatically variegated surround is in general not functionally equivalent to a homogeneous surround with identical chromatic average.

Furthermore, Lotto and Purves (2000) showed that increased numerosity may have opposite effects on color perception depending on the distribution of the surrounding chromaticities: Higher numerosity leads to enhanced perceived color differences between two identical patches, if the color distributions in the surrounding areas are consistent with two different illuminations (i.e., the identical input of the test patches has to be attributed to different reflectances). In contrast, higher numerosity leads to attenuated perceived color differences between those identical patches, if the color distribution is consistent with an identical (white) illumination in both surroundings (i.e., the identical input of the test patches has to be attributed to identical object color).

In summary, an increase in numerosity may improve the estimation of the illumination color for different reasons: Through an increasingly better estimation of the illumination color by the spatial average of stimulus colors, through an increased chromatic variance, which further restricts possible illuminations, or via additional higher order statistics of the input, which represent physical regularities of light and surface interaction that can be exploited by the visual system. However, in the achromatic domain, there is some evidence that the average luminance is not always the crucial factor. In particular, Gilchrist et al. (1999) reported that gamut compression (an indicator of constancy failure) in the staircase Gelb effect decreases with numerosity, even when the luminance range (and thus average luminance) is held constant.

The inconsistencies regarding an effect of numerosity on color constancy found in the literature may partly be due to a methodological problem: Many studies present simple 2D stimuli in isolation and it is in general unclear whether observers actually perceive a change in illumination as such, even if a constant scene geometry or the instructions may suggest so. Depending on whether or not a change in illumination is assumed, the perceived surface color and thus the result of an asymmetric color match can change drastically. If a uniform illumination covering both the test and the match stimulus is perceived, a proximal match should be performed, that is, a match of the tristimulus values. In most works on color constancy a proximal match is regarded as tantamount to zero constancy. If, in contrast, the change in illumination intended in the stimulus construction is actually recognized, a distal match is expected, which usually corresponds to the tristimulus values resulting from a physically correct calculation of the light reflected from the respective surface. It is possible that certain simple 2D configurations do not reliably trigger the perceptual interpretation intended in the stimulus generation or suggested in the instruction. Ambiguous findings with seemingly similar 2D stimulus material may thus at least partly be attributed to an instability of the perceptual modes they trigger. This assumption is supported by the fact that, in experimental setups with real illuminations and pieces of paper, in which the change of illumination was clearly recognizable, an effect of numerosity was frequently observed (Burzlaff, 1931; Henneman, 1935; Katz, 1930; Gilchrist et al., 1999).

Hence, it is not only the statistical distribution of the chromaticities, but also the spatial arrangement of the colors in the stimulus that plays an important role in the perception of surface lightness and color. In the next section, we consider stimulus configurations that range from local color contrasts between adjacent surfaces to figural aspects of the 3D position and orientation of surfaces in scenes.

#### Spatial organization

From the stimulus-centered perspective, the figural arrangement of homogeneously tinted patches in simple stimuli is often characterized by the local colors or the luminance ratios they contain and the distances of surround elements to the target field. Local contrast ratios between the target and the surround have been found to have an impact on lightness and color constancy. This seems reasonable, because the local (and global) contrast ratios between natural reflectances remain approximately constant under changes of natural (broadband) illuminations (Foster & Nascimento, 1994). Gilchrist et al. (1999), for example, found higher degrees of constancy with a Mondrian pattern than with a linear chain of five or 10 luminance patches. From a stimulus-centered perspective, this improvement could be explained by a greater number of different adjacent excitation ratios. However, because even simple 2D arrangements of color patches may trigger rather complex scene interpretations, it is also possible that grouping processes lead to a reduced number of distinguishable perceptual objects and thus to a qualitatively different color appearance. To test the contribution of such grouping processes, Schirillo and Shevell (2002) conducted a matching experiment in which they compared the effect of spatial organizations of 10 achromatic patches that were either consistent with a parsimonious interpretation of five surfaces under two different illuminants or not consistent. In the former case, that is, if the configuration supported the interpretation that the test and the comparison patches are located in different illuminations, the results were found to shift toward a reflectance match.

From a physical point of view, the distinction between an illumination edge and a reflectance edge requires a 3D scene interpretation, because the assumed light sources must be located somewhere in space and there must be something that prevents a homogeneous illumination of the entire stimulus area. Thus, even simple 2D mosaics of homogeneous color patches can provide cues not only about the illumination color, but also about spatial changes in illumination strength, for example, caused by (assumed) shadows, spotlights, transparent layers, or depth.

From the scene-centered perspective, such scene interpretations of the input play a major role and there is a large body of work addressing the extent to which the 3D interpretation of a stimulus determines the perceived illumination and/or vice versa. Many well-known cues, which the visual system could potentially use to create a 3D model of the outside world, have been suggested in the past and most of them have been investigated with respect to lightness and color constancy.

For instance, it was observed that the perceived surface color depends on the perceived orientation and depth of a surface (Adelson, 1993; Gilchrist, 1977; Gilchrist, 1980; Radonjić & Gilchrist, 2013; Schirillo & Arend, 1995; Wishart, Frisby, & Buckley, 1997; Yang & Shevell, 2002). Gilchrist (1977) varied the depth interpretation of a 3D scene by changing the viewing condition, while keeping the retinal input essentially constant. The observers judged the lightness of two gray test patches of identical luminance. When viewed binocularly, one of the patches was seen as coplanar to a white surface, the other as coplanar to a black surface. They were accordingly interpreted as grey (weakly reflective under bright illumination) and white (highly reflective under dim illumination). With monocular viewing, the perceived orientation and thus both the perceived contextual belonging and the perceived lightness of the two test patches were reversed, which suggests that the observers inferred the illumination of a patch from its perceived orientation (see also Radonjić & Gilchrist, 2013; Radonjić, Todorović, & Gilchrist, 2010).

There are also a number of experiments that found no (or only a very small) depth effect on lightness and color constancy (e.g., Gibbs & Lawson, 1974; Hurlbert & Wolf, 2004; Kraft et al., 2002; Schirillo, Reeves, & Arend, 1990; Schirillo & Shevell, 1993). One explanation for these inconsistent results could be that the studies without an effect failed to provide sufficient (image) cues to evoke the intended interpretations regarding the geometry and illumination of the scene (cf. Gilchrist, 1977). Boyaci, Maloney, and Hersh (2003) controlled for the actually perceived surface orientation when measuring the perceived surface color. They used rendered 3D scenes that contained many cues for surface depth and for the position of a punctual light source (e.g., stereo disparity, cast shadows, and specular highlights) and found reliable effects of the perceived surface orientation on the perceived surface color. This finding supports the assumption that the perceived position and orientation of a surface relative to the perceived illumination direction is crucial for lightness and color constancy.

Together, these results suggest that the mechanisms underlying surface color perception refer to internal representations of the illumination pattern in the inferred 3D scene. Further evidence in support of an approach that emphasizes the special role of illumination perception in color constancy is provided by the finding that constancy is increased in scenes that contain specular highlights (e.g., Snyder, Doerschner, & Maloney, 2005; Yang & Shevell, 2002) and mutual reflectance (Bloj, Kersten, & Hurlbert, 1999).

#### Spatial configuration and numerosity in real scenes

Studies that used naturalistically rendered scenes or real scenes, which usually include cues for spatial variations of illumination intensity and color, often observed high degrees of color constancy (e.g., Brainard, Brunt, & Speigle, 1997; De Almeida, Fiadeiro, & Nascimento, 2004; Foster, Amano, & Nascimento, 2006; Kraft & Brainard, 1999; Radonjić, Cottaris, & Brainard, 2015a; Xiao, Hurst, MacIntyre, & Brainard, 2012). Radonjić et al. (2015a) report that the high level of constancy found with naturalistic stimuli and stereoscopically evoked depth was greatly reduced when these stimuli were simplified to flat color patterns, although the chromaticity and luminance distributions of the background colors were highly similar in both types of stimuli. Kraft and Brainard (1999) used natural scenes with actual illuminated surfaces and found a high degree of constancy in their physically plausible control condition with identical reflectances under different illumination (CI = 0.83). A significantly decreased constancy (CI = 0.39) was found if reflectances and illumination were adjusted in such a way that the mean color in the entire stimulus was identical across the two conditions with different illumination. This result is plausible, because this manipulation counteracts the rule that an increase in the number of random samples from a fixed population of reflectances leads to a more accurate estimate of the illumination color by the spatial average of the scene colors. Kraft, Maloney, and Brainard (2002) found that increasing the numerosity of a real scene by adding a Macbeth Color Checker Chart had no effect if an illumination change was combined with a fixed reflectance of the wall and additional objects (*valid cue condition*). However, increasing the numerosity led to improved constancy in an *invalid cue condition*, in which the reflectance of the wall was exchanged and the chromatic mean value did not provide valid information about the illumination change. This difference in the effectiveness of numerosity might be explained by a ceiling effect in the valid cue condition and by a more valid estimation of the illumination with the auxiliary cues of the Macbeth Color Checker Chart in the invalid cue condition.

The effect of numerosity on the degree of constancy seems to depend on the availability of convincing depth cues. Radonjić and Gilchrist (2013) report a positive effect of high numerosity for monocular stimulus presentation but not for binocular presentation. Furthermore, in simulated 3D scenes Radonjić et al. (2015b) found no advantage of using variegated floor and wall surfaces instead of homogenous ones. Even in real 3D scenes, constancy was just as high with a uniform surround as with 24 additional colored objects in the scene (Nascimento, De Almeida, Fiadeiro, & Foster, 2005). All three works may indicate a ceiling effect: Once enough other cues such as stereodisparity, cast shadows, penumbrae, object shading, and so on, are available to construct a consistent illumination pattern of the scene, the number of surface colors could play a less decisive role. This interpretation is supported by the observation that also other cues sometimes fail to have an additional effect: De Almeida, Fiadeiro, and Nascimento (2010) found that constancy with realistic 3D scenes was just as high as in a modified “2D” version, in which the shading of the 3D objects was selectively removed (average value of CI = 0.82). This finding indicates that the shading of the 3D objects had no effect beyond that already observed with the remaining illumination and depth cues in the surround.

Accordingly, the absence of an effect of numerosity would be expected whenever increasing numerosity does not contribute to a valid illumination estimate. This would be the case, for example, if the color distribution in the stimulus indicates an illumination that differs from the true illumination used in stimulus generation or if sufficient other valid illumination cues are available.

However, in some studies with realistic scenes, the degree of observed constancy was surprisingly low. Robilotto and Zaidi (2004), for example, found that observers could not reliably identify real objects of the same reflectance across different illumination levels, that is, low lightness constancy. Although we are sensitive to physical changes in the illumination to some extent, such results indicate that our ability to perceive constant surface colors is limited. This may reflect objective limits, for example, owing to metamerism (e.g., Logvinenko, Funt, Mirzaei, & Tokunaga, 2015). Some studies suggesting a very restricted ability to estimate the illumination even raise serious doubts about the illumination estimation hypothesis to explain color constancy (Granzier, Brenner, & Smeets, 2009). One has to concede that it is currently not clear in which situations we make illumination estimates and how veridical such estimates are. Nevertheless, recent work on material recognition indicates that the use of realistic illumination maps or light fields and physically correct image generation can have a major impact on perception (e.g., Dror, Willsky, & Adelson, 2004; Fleming, Dror, & Adelson, 2003; Faul, 2019).

It is evident that the naturalness of a scene has been described and classified on very different dimensions, which cannot easily be fitted into a simple taxonomy. At the feature level, it is possible to describe the stimuli in terms of color and geometry. Such features, in turn, have been considered either from a stimulus-centered perspective, which emphasizes statistical properties of the stimulus, or from a holistic scene-centered perspective, which focuses more on the meaning of the stimulus. The explanations proposed for the effect of the naturalness of stimuli on the degree of lightness and color constancy refer to both low-level and high-level mechanisms.

In summary, the available evidence indicates that the perception of surface colors in natural scenes is significantly more constant, that is, less influenced by the viewing context, than in simple abstract scenes. The partially inconsistent findings regarding the degree of constancy in natural scenes suggests that our knowledge about relevant regularities in the input and the underlying mechanisms remains incomplete.

### Articulation and transparency

We recently conducted a first investigation of transparent layer constancy in naturalistic rendered scenes (Falkenberg & Faul, 2019b). In an experimental setup similar to the one used by Radonjić et al. (2015a), we compared the transparent layer constancy for flat transparent objects in rendered complex scenes with that observed in color-matched simple color mosaics and found an enhanced transparent layer constancy in the complex scenes. The relevant cues and mechanisms underlying this enhancement have not yet been identified.

Here we consider the role of numerosity for transparent layer constancy. We have already briefly summarized how the influence of the naturalness of the scene on the degree of constancy has been investigated in the domain of object lightness and object color. However, it is unclear if the findings of such studies can be generalized to the transparent filter case where changes in the background do not only affect the neighborhood of the filter region, but also the colors seen through it. In a sense, articulation is a prerequisite of perceptual transparency: Against a homogeneous background, even a light-transmitting object like a piece of stained glass may appear opaque. Another essential difference to color constancy is the depth information that is inherent even in very simple stimuli in which a transparent layer is perceived.

There exist only a few studies on the role of articulation for the perception of transparent layers. Gerardin, Roud, Süsstrunk, and Knoblauch (2006) observed an enhanced impression of transparency when they increased the number of squares in the checkerboard patter that was used as background. Here, numerosity is solely defined geometrically as the number of distinct patches, because the same two colors were used in the minimal bipartite and the multipartite stimuli. Numerosity can also be defined as the number of different reflectances or chromaticities in the stimulus. Because the number of geometrical and chromatic surfaces often coincide, these two meanings were usually not differentiated explicitly.

Faul and Ekroll (2012) informally observed a greater homogeneity of the perceived filter properties when a greater number of different background colors were superimposed by the filter. Furthermore, they observed high degrees of scene constancy, that is, the perceived properties of a transparent layer were hardly affected by changes in 10 background reflectances.

In Falkenberg and Faul (2019a), we tested the numerosity hypothesis explicitly by varying the texture density of the background and did not find an effect. We argued that this outcome may be due to a ceiling effect, that is, that already the lowest texture density contained a sufficiently large number of background colors. To counteract such a ceiling effect in the present investigation, we investigate the influence of numerosity with a significantly lower number of colors per scene.

## Experiment 1: Random color selection

The purpose of this experiment was to test the hypothesis of a positive effect of numerosity for transparent layer scene constancy. Numerosity is varied in two levels. The lower level comprises 2 background colors, which represents a minimal stimulus for the examination of a transparent layer, and the higher level comprises 10 background colors. We expect the critical number of colors to lie within this range, because Faul and Ekroll (2012) observed very high degrees of transparent layer scene constancy with 10 color patches. The relative degree of constancy is determined by comparing the errors in a filter-matching task made with backgrounds containing 2 versus 10 randomly drawn background colors from a fixed population of reflectances. Because we are interested in scene constancy, only the object reflectances in the test stimulus are exchanged while the illumination is held constant. This corresponds with a situation in which a filter object moves in front of a homogeneously illuminated scene.

### Methods

#### Stimuli

We simulated a standard scene that comprises 162 reflectances that were illuminated by CIE daylight with a color temperature of 6500K and led to color codes within the gamut of our monitor (EIZO ColourEdge CG243W 24.1 in, 1920 × 1200 pixel, 60 Hz refreshing rate, controlled by a NVIDIA Quadro 600 graphics card with a bit-depth of eight bits per channel; the monitor spectra used in the calibration of the monitor were measured with the spectroradiometer JETI specbos 1211). The background reflectances were randomly chosen frequency-limited spectra (Stiles, Wyszecki, & Ohta, 1977) with a limiting frequency of 1/120 cycles/nm, which were rescaled to a range from 0.0 to 0.6. The resulting smooth and broadband reflectance functions are considered representative for natural reflectance spectra. The light reflected from the background was computed as the pointwise product of the background reflectance and the daylight spectrum and then transformed to LMS coordinates (Stockman, MacLeod, & Johnson, 1993) for further calculations.

**Figure 1.**
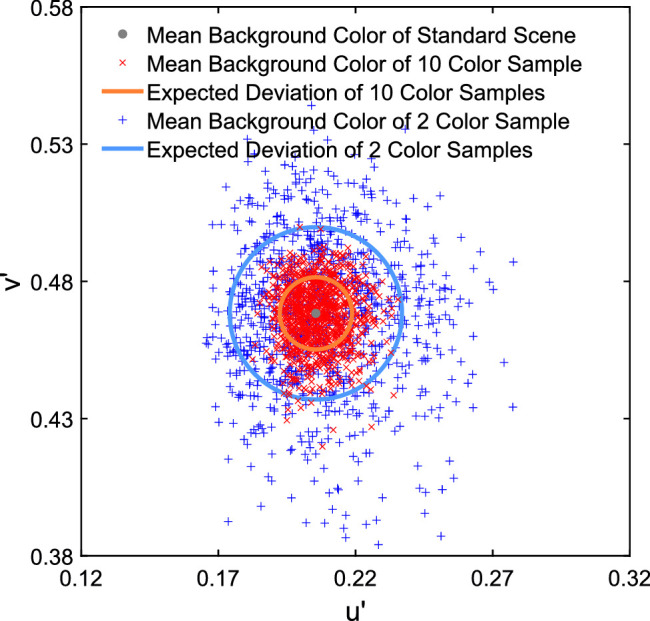
Distributions of the chromatic mean for the 2 color subsets (blue plus) and the 10 color subsets (red cross) randomly drawn from the 162 colors of the standard scene.

**Figure 2.**
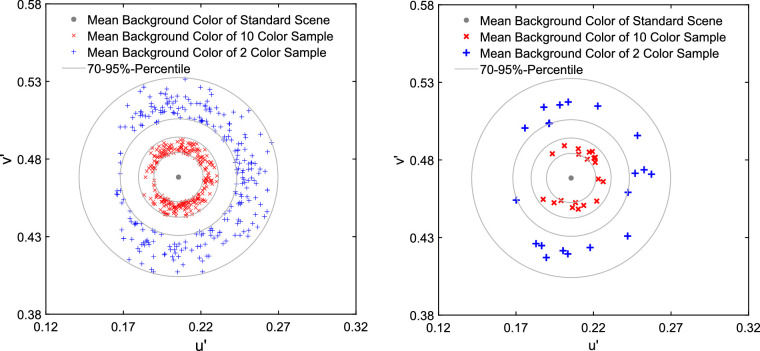
The left panel shows all drawn subsets with a mean color lying between the 70th and 95th percentiles. From this pool, 20 subsets for each of the two levels of numerosity were randomly drawn (right panel).

To decide which specific color subsets of the standard scene should be tested, we analyzed the distributions of samples with 2 and 10 colors drawn from the total population of 162 colors. For this purpose, 1000 subsets were drawn randomly for each numerosity level. Naturally, the chromatic averages of 2 color subsets show a stronger variation than the chromatic averages of subsets that contain 10 colors, which on average are closer to the overall mean of the standard scene (Figure 1). To reflect the different expected deviations from the chromatic average of the standard scene for both types of subsets (2 vs. 10 background colors), we randomly picked 20 subsets for each type of subset from the 70% to 95% percentiles of the respective subpopulation (Figure 2).

**Figure 3.**
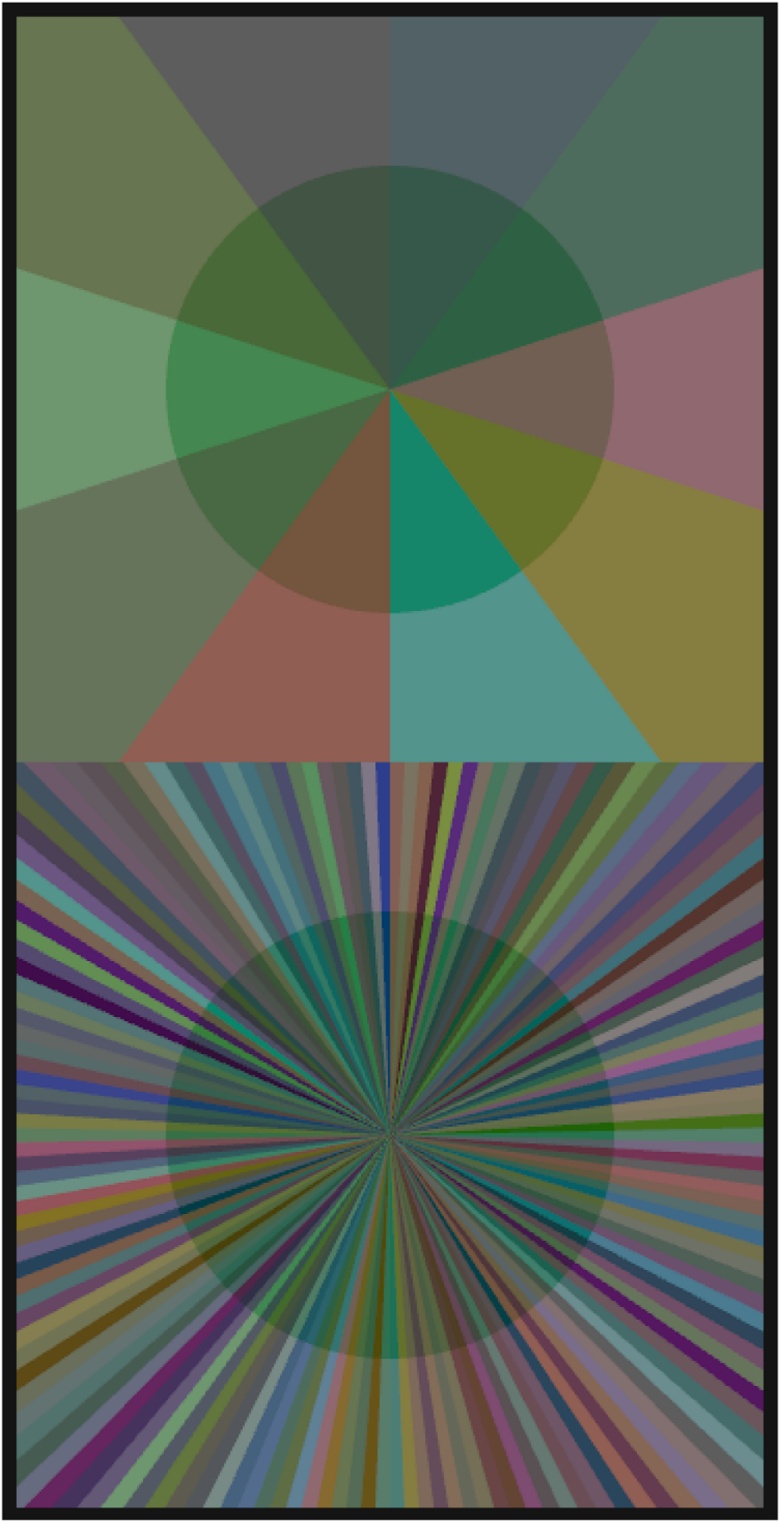
The general stimulus layout for the green clear filter. The upper half of the stimulus is the test scene in which the filters are presented in front of different subsets. In this case, a subset of 10 randomly drawn colors from the 162 colors of the standard scene. The lower half of the stimulus is the standard scene in which the matching is performed. The 162 colors of the standard scene were randomly ordered in each trial.

The test stimulus is a simple scene composed of either 2 or 10 surfaces with the colors of the chosen subsets (see Figure 3 for the general stimulus layout). To ensure that each exposed background color and the corresponding color seen through the filter are linked unequivocally, the different reflectances were arranged in a pie chart with an equal arc length of each sector. The same applies to the standard scene in which, owing to the larger number of reflectances, the opening angle of each sector was significantly smaller. The test scene and the standard scene were presented one above the other without a gap resulting in a total stimulus width of 13.9° and a height of 27.4°. To evoke a vivid percept of a transparent layer floating in front of the background, the scenes were presented stereoscopically with the respective filter regions shifted nasally by 4.86 mm for each eye. The diameter of the circular filter objects was 8.3°. The observers viewed the stimuli from a distance of 400 mm through a mirror stereoscope (SA200 Screenscope Pro).

**Figure 4.**
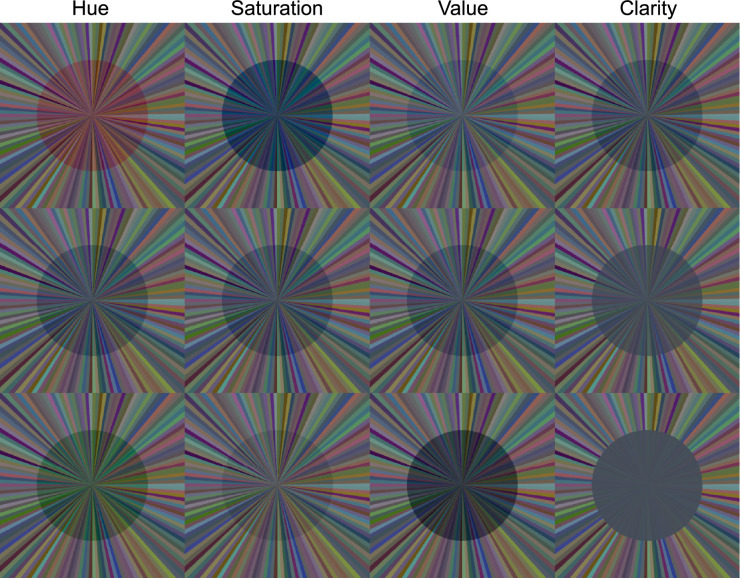
Visualization of the four perceptual filter parameters according to the model of Faul and Ekroll (2011). First column: Hue variations of a greenish, a bluish, and a reddish filter. Second column: Saturation variation from high saturation (top) to low saturation (bottom) for the bluish filter. Third column: Transmittance variation from high value (top), which corresponds with a high overall transmittance, and low value (bottom), which corresponds with a low overall transmittance. Fourth column: Clarity variation from a clear filter with no direct reflection (top) to a hazy filter with strong direct reflection (bottom).

To calculate transparent layers covering the background surfaces, we used the perceptual filter model suggested by Faul and Ekroll (2002, 2011), according to which a transparent layer can be described by the perceptual dimensions of hue, saturation, overall transmittance (i.e., value), and clarity (see Figure 4 for the perceptual dimensions of the filter parameters). We applied the proposed parameterization of Faul (2017) in which the formerly nominal parameters HSVC are transformed into an approximately perceptually uniform parameter space for filter transparency (UHSVC) by an invertible transformation. The advantage of using this uniform filter parameter space is that it facilitates the navigation through the four-dimensional space owing to the increased independence between the four parameters and the perceptual equidistance within each scale. Three different filter hues corresponding to a green, blue, and red layer (hue [H*] = 0.39, 0.72, and 0.98, respectively) were chosen with a saturation corresponding to 35% of the maximal realizable saturation of the respective hue (saturation [S*] = 0.30, 0.28, and 0.37, respectively). Clarity (C*) was varied in two levels, namely clarity = 1.0, which corresponds with a clear filter without direct reflection, and clarity = 0.49, which corresponds with a filter of medium haziness. The overall transmittance was held constant for each filter hue (value [V*] = 0.96, 0.84, and 0.93, respectively), independent of the clarity level. The chosen filter parameters were then applied to the background colors of both the standard and the test stimulus to compute the final filter colors (see Appendix A, Equation A.1).

The combination of 20 test subsets × two levels of numerosity (2 vs. 10) × three levels of filter hues × two levels of filter clarity resulted in 240 trials. We conducted five control trials for each of the six investigated filters (30 trials), in which the 162 background colors of the standard scene were also present in the test stimulus (symmetric match). This was done to estimate the maximal achievable accuracy in the matching task for each participant. Additionally, all scenes containing a blue filter were recorded twice per participant as an exemplary test of the reproduction reliability of the adjustment procedure for one of the three filters (80 trials). This finally led to 350 trials per participant.

#### Procedure

**Figure B1.**
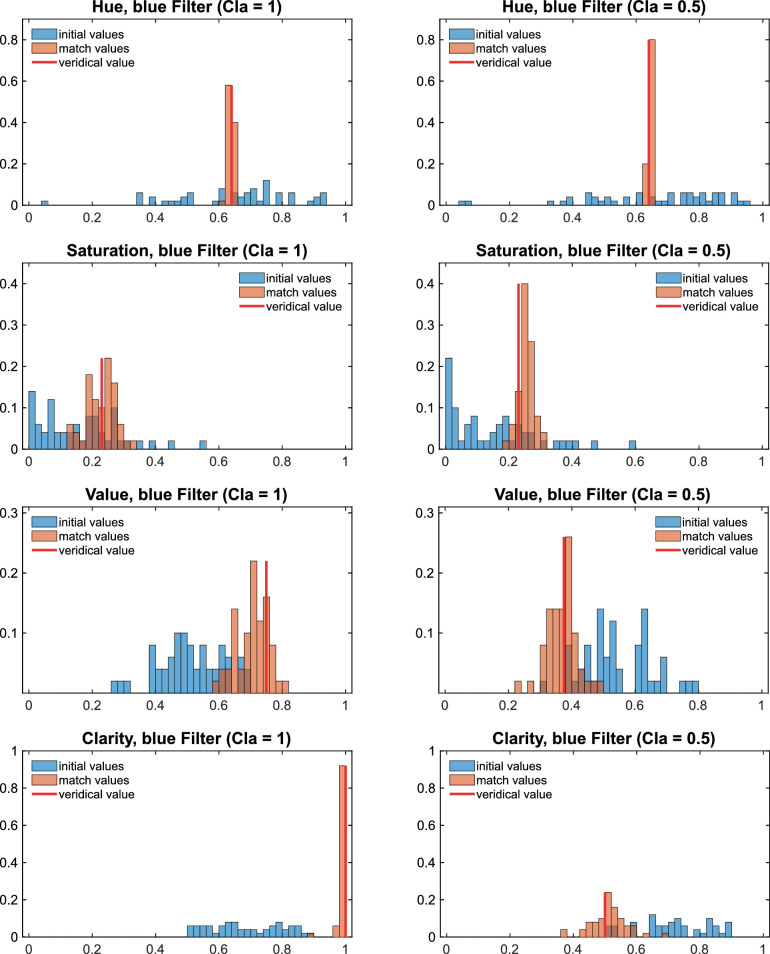
Distributions of the observed settings in the HSVC parameter space for clear (clarity = 1.0) and hazy (clarity = 0.5) blue filters in the control trials (red bars) of Experiment 1 and Experiment 2 (2 × 5 participants × 5 repeated measurements) and the veridical value of the respective filter parameter in the test scene. The second distribution (blue bars) refers to the initial values in these trials.

**Figure B2.**
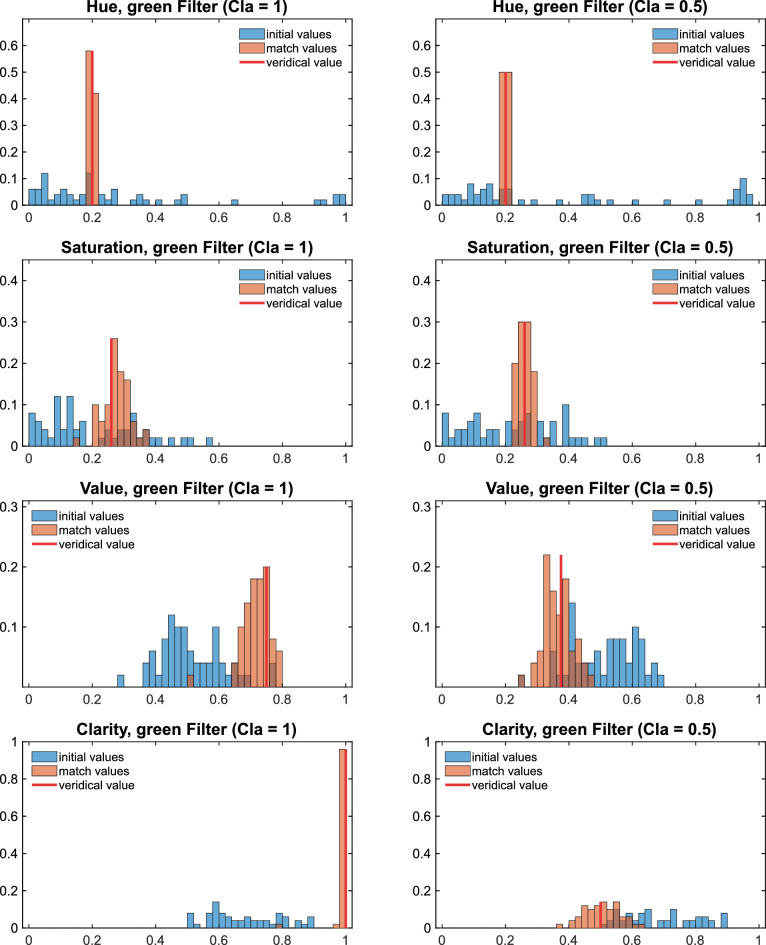
Distributions of the observed settings in the HSVC parameter space for clear (clarity = 1.0) and hazy (clarity = 0.5) green filters in the control trials (red bars) of Experiment 1 and Experiment 2 (2 × 5 participants × 5 repeated measurements) and the veridical value of the respective filter parameter in the test scene. The second distribution (blue bars) refers to the initial values in these trials.

**Figure B3.**
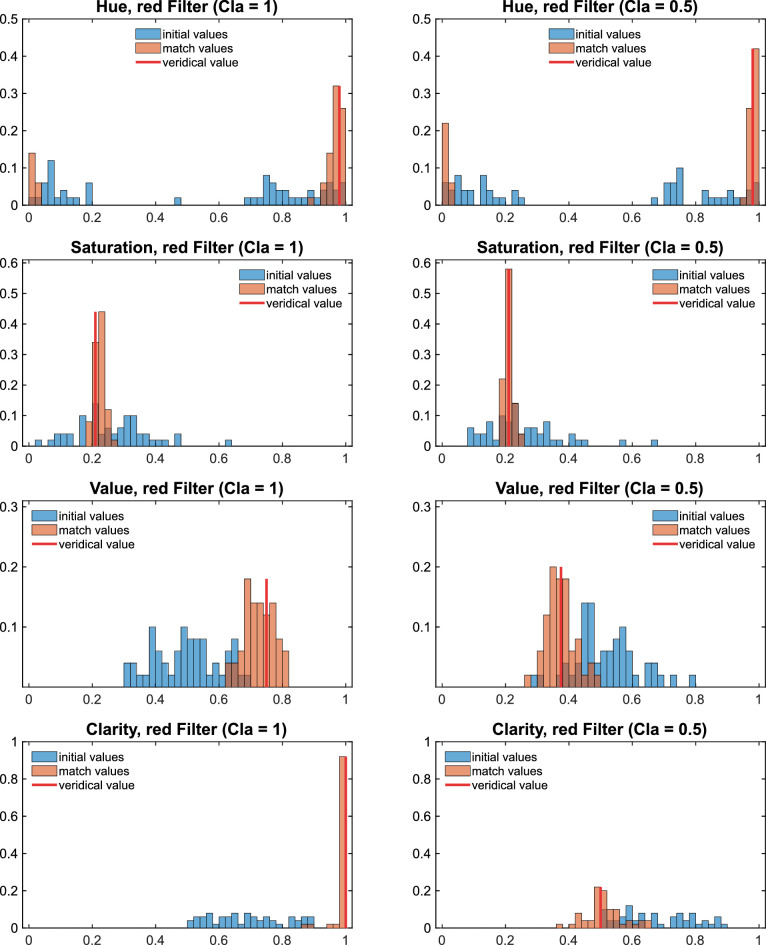
Distributions of the observed settings in the HSVC parameter space for clear (clarity = 1.0) and hazy (clarity = 0.5) red filters in the control trials (red bars) of Experiment 1 and Experiment 2 (2 × 5 participants × 5 repeated measurements) and the veridical value of the respective filter parameter in the test scene. The second distribution (blue bars) refers to the initial values in these trials.

The participants’ task was to adjust the four parameters—hue, saturation, value, and clarity—of the filter in the standard scene so that it appears to be the same filter as shown in the test scene. In each trial, the initial values of the parameters in the standard filter deviated randomly from the filter parameter of the test scene but it was always ensured that the filter was displayable (Figures B1–B3 in Appendix B show the distribution of the initial parameter values for the symmetric control trials). The participants could adjust each parameter one by one in the range from 0 to 1 both in large (±0.010) or small (±0.001) steps by pressing the up and down or the left and right arrow keys on the keyboard, respectively. The space key was used to switch between the four parameters at will. For the noncircular parameters—namely, saturation, value, and clarity—an acoustic signal indicated that the minimum or maximum value was reached. Because such four-dimensional settings require some practice to be able to navigate properly in the parameter space, the participants performed five exercise trials before the actual experiment. There was no time limit for the settings. After the best possible setting of the filter parameters has been made, the participants rated the quality of the match on a five-level scale where 1 denotes a very poor match (looks like a different object with no similarity to the test filter) and 5 a perfect match (looks like the same filter as presented in the test stimulus). If participants noticed they made a mistake, they had the opportunity to mark a trial as invalid.

#### Participants

Five undergraduate students took part in the experiment, all of them naïve regarding the purpose of the experiment. The experiment was conducted in five or six sessions lasting about 1.5 to 2.0 hours each. All participants had normal color vision, as assessed by the Ishihara plates (Ishihara, 1967), and reported normal or corrected-to-normal visual acuity. Participation was voluntary and written informed consent was obtained.

### Results

The evaluation of the observed filter settings will first be conducted in the uniform filter parameter space (UHSVC). Owing to the approximate perceptual equidistance of the scales hue (H*), saturation (S*), value (V*), and clarity (C*), it is meaningful to consider mean values and variability in this parameter space (Faul, 2017). The low overall variance in the control trials (i.e., symmetrical matches), in which the six investigated test filters were presented in the standard scene, indicates that the participants were able to navigate the parameter space and to perform accurate settings independent of the random starting values of the filter parameters (see Figures B1–B3 in Appendix B). To further test the reliability of the observers’ settings, each of the 20 subsets of background colors has been measured twice for all conditions with the blue filter. Because the variance of the two repeated measurements was comparably low compared with a simulation of an equal number of repeated measurements in the symmetric control trials for each observer, the average value of the double measurements was used in subsequent analyses. This process leads to a final dataset of 1200 measurements in the experimental condition, minus three measurements that were marked as erroneous by the participants and therefore excluded from the analysis.

**Figure 5.**
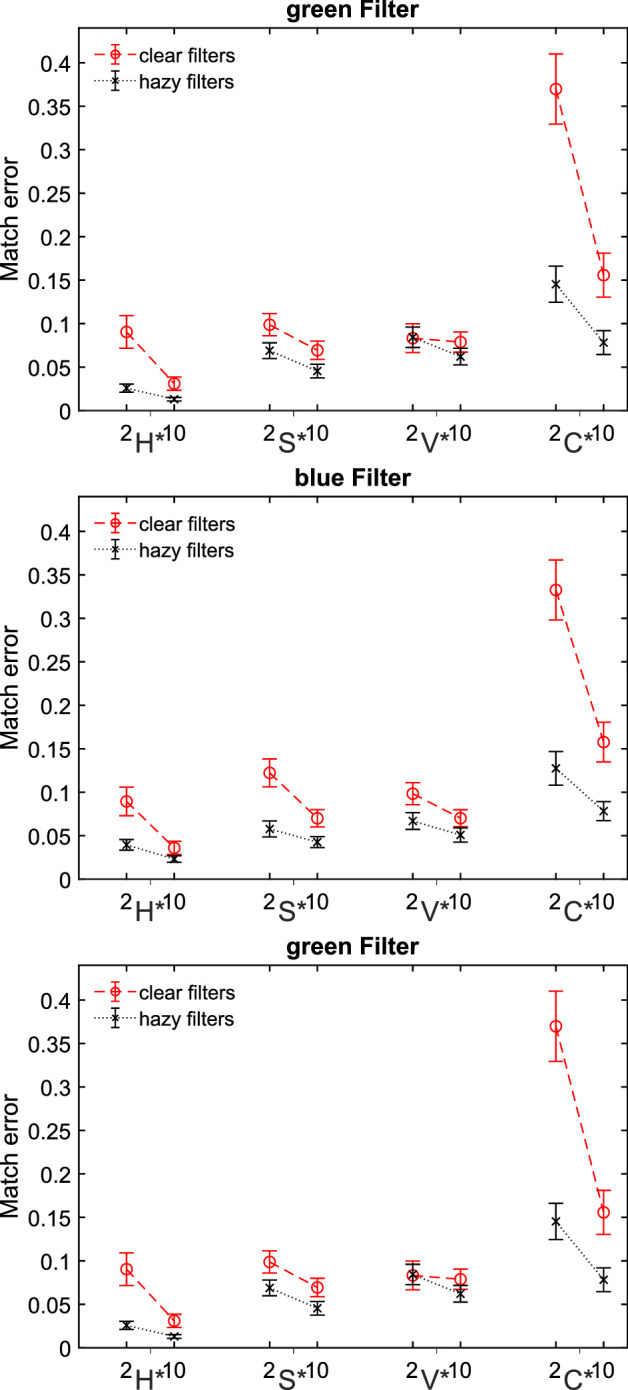
Deviation of the uniform parameters hue (H*), saturation (S*), value (V*), and clarity (C*) from the values of the green (top), blue (middle), and red (bottom) filter in the test scene for subsets of 2 versus 10 background colors. Each data point is the mean of the absolute error across 100 observations (20 test scenes × 5 participants) minus occasional exclusions. Error bars indicate ± 2 *SEM*.

Figure 5 shows the observed settings for the four parameters of the filter model in the experimental condition. The deviations of the matched parameters from the parameters of the test filter show the same pattern for all three filter colors: Basically, smaller deviations (i.e., smaller match errors) were found for scenes with 10 background colors than for scenes with 2 background colors. In addition, significantly smaller deviations were generally observed for hazy filters than for clear filters.

**Figure 6.**
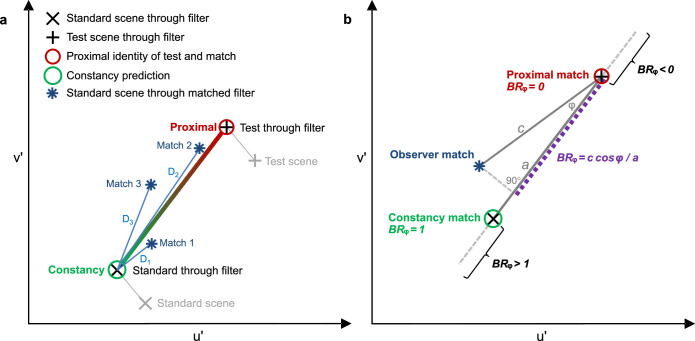
(A) The two theoretical poles of filter matches in the standard scene the constancy prediction according to our perceptual filter model (green circle) and the proximal identity of the filter region of a match in the standard scene and the test scene (red circle). The constancy prediction refers to the mean color in the filter region of the standard scene (grey cross) shifted in color space when seen through the identical filter as presented in the test scene (black cross). Proximal identity refers to a mean color in the filter region of the standard scene that is identical to the mean color of the test scene (grey plus) seen through the respective test filter (black plus). Matches 1 to 3 (blue asterisks) show a match that strongly corresponds with the constancy prediction (Match 1), one that strongly corresponds with the proximal identity criterion (Match 2), and one that considers both criteria with approximately equal weight (Match 3). D_1_ to D_3_ are the respective Euclidean distances, as a measure of the absolute deviations of the matches from the constancy prediction. (B) The calculation of the projected Brunswik ratio (*BR*ᵩ) as a relative measure of constancy (length of violet dashed line). A constancy match that would correspond to the constancy prediction would have a *BR*ᵩ of 1. A proximal match would show the identical mean color in the filter region as in the presented test scene and would have a *BR*ᵩ of 0.

**Figure B4.**
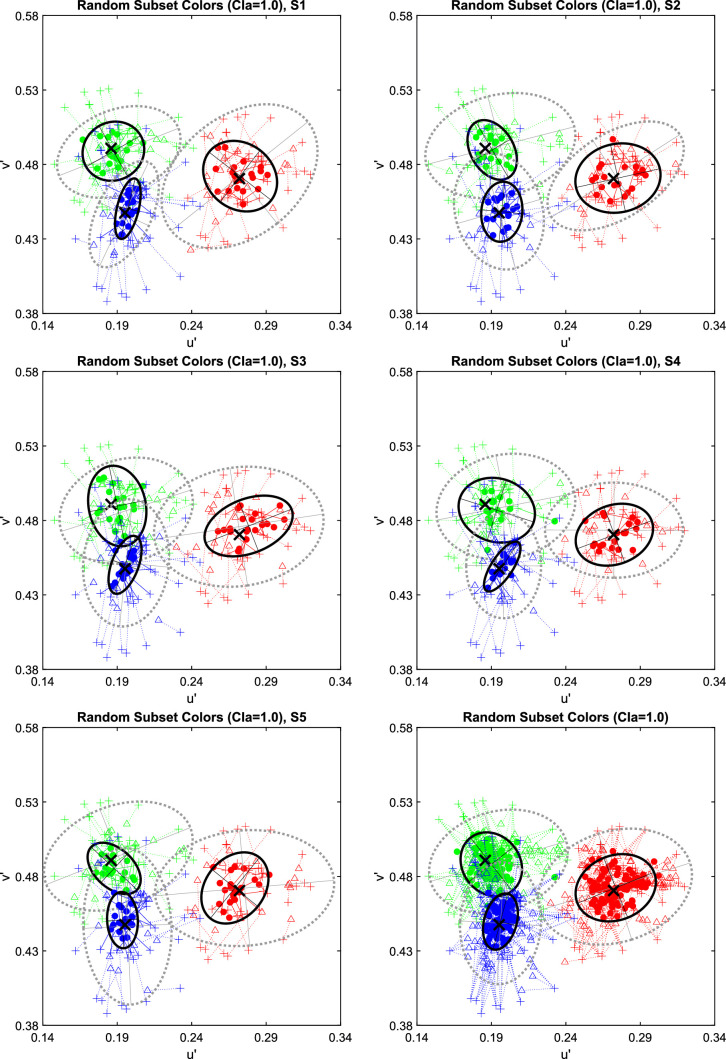
Matching results of the clear filters (clarity = 1.0) in Experiment 1 plotted separately for each participant. The lower right panel contains the data of all observers. The ellipses indicate the 95% CI for the 2 color subsets (dashed line, open triangle) and 10 color subsets (solid line, filled circle). See also the legend of Figure B5.

**Figure B5.**
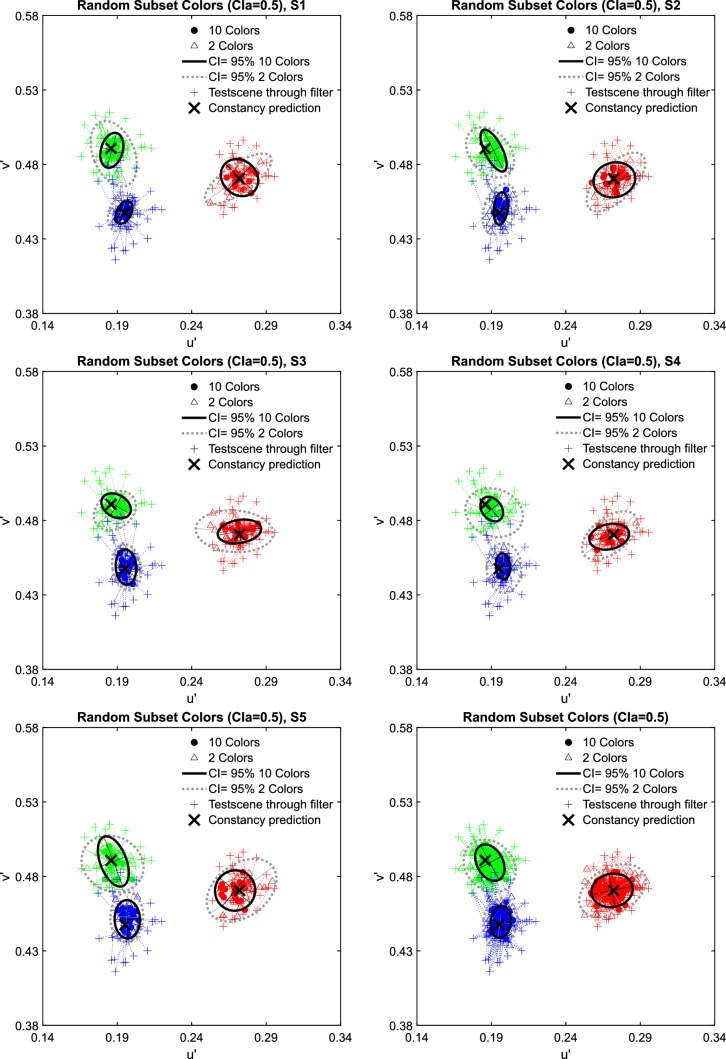
Matching results of the hazy filters (clarity = 0.5) in Experiment 1 plotted separately for each participant. The lower right panel contains the data of all observers.

According to the numerosity hypothesis, a greater number of color patches in the test scene should increase the degree of constancy. Plotting the data in the u'v'-chromaticity space is suitable to visualize the mean color in the filter region of the standard scene at both perfect constancy according to our perceptual model and proximal identity to the test scene, and to consider the observed filter settings in terms of their location with respect to these two theoretical poles. Figure 6 shows the logic of this consideration and explains two common measurements of the degree of constancy that we subsequently refer to. First, the absolute distance of the matched filter settings from the constancy prediction in u'v'-chromaticity space (Figure 6a), and second, the projected Brunswik ratio (*BR*ᵩ) as a relative measure of constancy (Figure 6b). To calculate the colors in the filter region of the observed matches, the UHSVC filter parameter settings are transformed into *τ* and *δ* (for transformation routines from UHSVC to HSVC see Faul, 2017, and from HSVC to *τ* and *δ* see Faul & Ekroll, 2011) and applied to the background colors according to Equation A.1 in Appendix A. Because the pattern was highly comparable between all five participants, the mean chromaticities were calculated across all participants for each of the 20 test scenes (plots of each observer's results and the raw data, including scatter ellipses, can be found in Figures B4 and B5 in Appendix B). This logic refers to the concept of a “phenomenal regression to the real object” (Thouless, 1931a, 1931b). Possible reasons why the phenomenal impression may deviate from the constancy prediction even if the latter could be estimated veridically from the stimulus are discussed in detail in Faul and Ekroll (2012). One of the reviewers pointed to an alternative interpretation of such a compromise in terms of anchoring with respect to the relevant field (local anchoring) or the foreign field (global anchoring) (for a detailed description of these concepts see, for example, Gilchrist, 2006).

**Figure 7.**
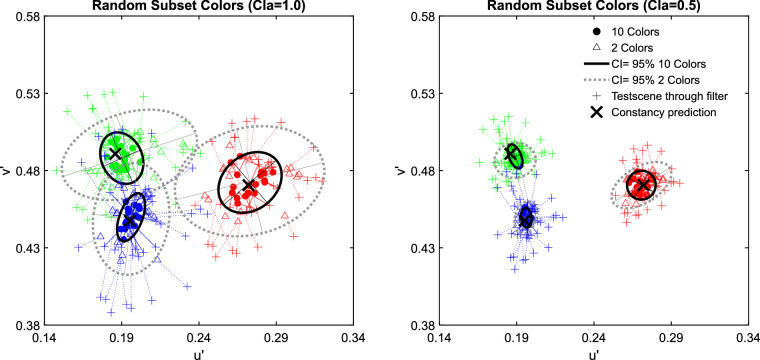
The results of the matching task in Experiment 1 for all three hues of the filter plotted separately for clear filters (left panel, clarity = 1.0) and hazy filters (right panel, clarity = 0.49). The small pluses indicate the positions of the mean color in the filter region for each subset presented as a test scene that is, the test scene seen through the respective filter and corresponds to the position where a proximal match would lie. The observed match for each subset is connected to the respective proximal match by a dotted line. Each data point corresponds to the chromaticity of the mean filter parameter setting for each subset across all five observers. The matches for the 10 color subsets are marked with filled circles and the matches for the 2 color subsets are indicated by open triangles. The constancy prediction is indicated with a black cross. The ellipses represent a 95% confidence interval (CI) for the observed matches.

**Figure 8.**
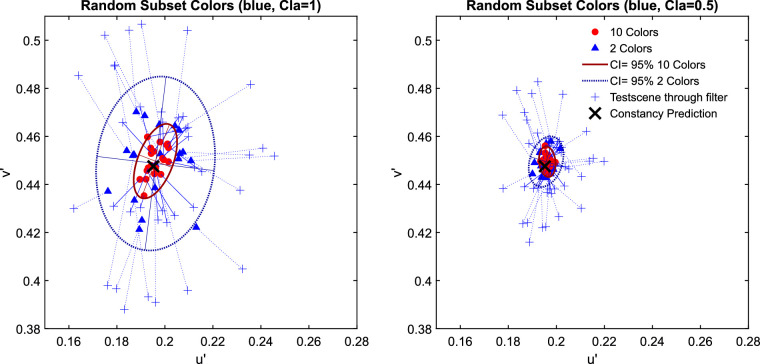
An enlarged view on the data for the blue filter in Figure 7.

**Figure B6.**
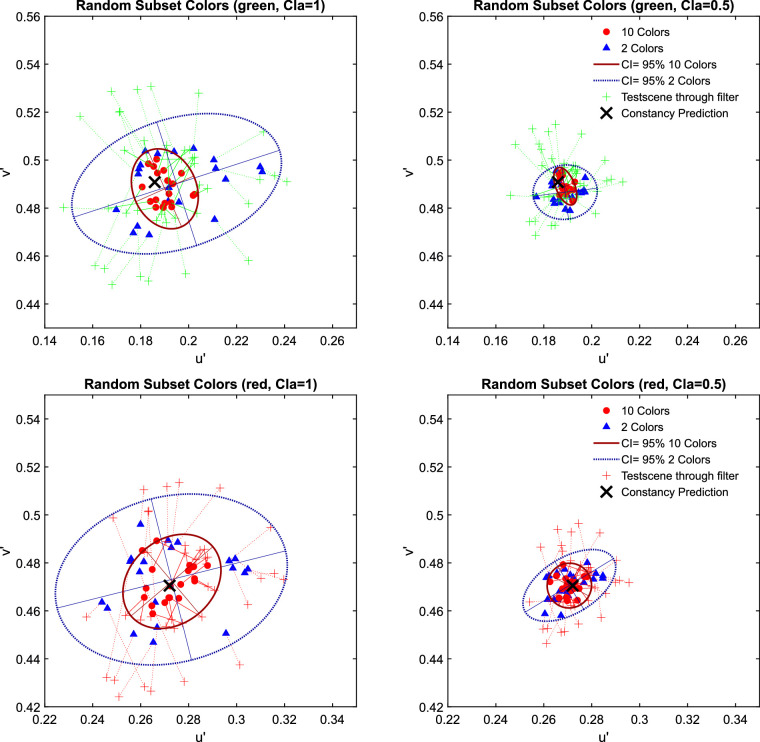
An enlarged view on the matching results of the green (top) and red (bottom) filters in Experiment 1. See the caption of Figure 7 in the Results section of Experiment 1 for details.

The examination of the data in the u'v'-chromaticity space suggests that higher numerosity leads to higher degrees of transparent layer scene constancy. For all three investigated filter hues, the absolute deviations from the constancy prediction are significantly smaller with 10 background colors than with only 2 (Figure 7). Figure 8 shows exemplarily for the blue filter the chromaticities of the mean settings across all five observers in more detail (the results for the red and green filter are similar and can be found in Figure B6 of Appendix B).

**Figure 9.**
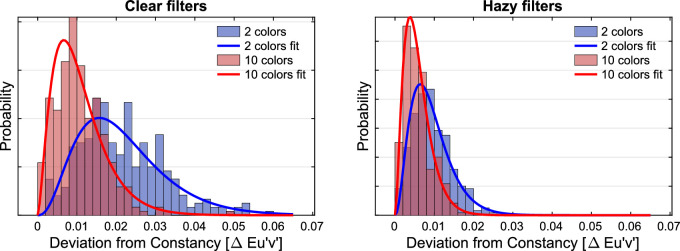
Distributions of the deviations of the matches from the constancy prediction in u'v'-chromaticity space depending on the numerosity of the test scene plotted separately for clear filters (left) and hazy filters (right). Each distribution refers to 300 measurements (minus occasional exclusions).

For each individual filter setting of the experimental condition, the Euclidean distance to the constancy prediction was calculated in u'v'-chromaticity space. The distributions of these deviations and their dependence on the numerosity of the subset as well as on the filter clarity are shown in Figure 9. To facilitate the comparison with later regression analyses in the results section of Experiment 2, a simple linear regression was performed. It indicates that numerosity is indeed a suitable predictor for transparent layer scene constancy: The lower the number of color patches in the test stimulus, the greater the deviation of the observed filter parameters from the constancy prediction (*r* = −0.39, *R*^2^ = 0.155, *df* = 1195, *p* < 0.001). The effect of numerosity is evident in both levels of filter clarity, but it is more pronounced for clear filters (*r* = −0.53, *R*^2^ = 0.276, *df* = 597, *p* < 0.001) than for hazy filters (*r* = −0.38, *R*^2^ = 0.144, *df* = 596, *p* < 0.001). In general, we observed higher degrees of transparent layer scene constancy for hazy filters than for clear filters (Figure 9).

**Figure 10.**
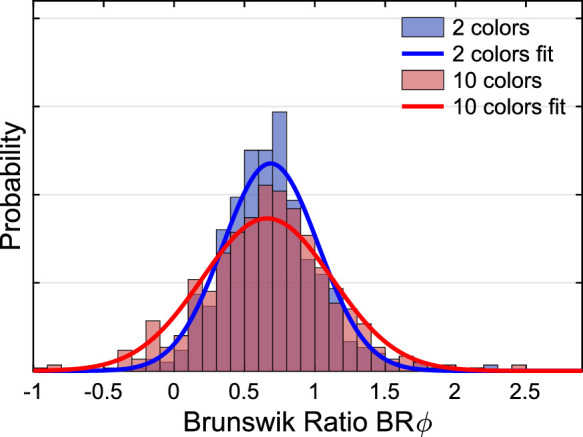
The distribution of the projected Brunswik ratios (BRᵩ) observed in the 2 and the 10 random color subsets in Experiment 1.

The observed u'v'-chromaticities may be used to compute an index for the degree of constancy. To this end, the Euclidean distance between the observed filter match and the constancy prediction (i.e., a filter with the same filter parameters as the one in the test scene) is set in relation to the distance between the constancy prediction and proximal identity (i.e., identical mean color in the filter area in the standard scene as in the test stimulus). In this case, the calculation of the Brunswik ratio is useful, because the index can also take values smaller than 0 and larger than 1, and the position of the settings in relation to the constant match is known. The filter settings in u'v’-chromaticity space are projected onto an imaginary straight line through the points corresponding with a proximal match and a constancy match (see Figure 6 for details). This projected Brunswik ratio (BRᵩ) reduces the overestimation of constancy and is described, for example, in Foster (2011). This relative measure indicates comparable degrees of transparent layer constancy in the 10 and the 2 color subsets (Figure 10).

### Discussion

In this experiment, random samples of 2 and 10 colors were taken from a fixed population of reflectances defined in a standard scene, and we found significantly smaller deviations of the perceived filter properties from the constancy filter with 10 color subsets than with 2 color subsets. This positive effect of numerosity is consistent with a number of findings from object color constancy. A further finding of this experiment is that, for both levels of numerosity, higher degrees of filter constancy were observed for hazy filters than for clear filters. This finding raises the question as to what the causes for this effect are.

One explanation for the greater degrees of transparent layer constancy with more background colors could be that adding more colors also increases the number of X-junctions, which is known to lead to a stronger (Gerardin et al., 2006) and more homogeneous (Faul & Ekroll, 2012) transparency perception. Combined with the finding that a more pronounced perceptual segregation of the filter from the background supports transparent layer constancy (Falkenberg & Faul, 2019a), this would predict the observed effect. However, the results of a small experiment reported previously (Falkenberg & Faul, 2017) speaks against this hypothesis. In that experiment, we increased only the number of surfaces (two vs. 10) while keeping the number of colors constant (two colors) and found no effect of increased numerosity on the degree of transparent layer scene constancy. Another argument against the plausibility of the better separation hypothesis is that the binocular disparity in the filter region already led to a very clear perceptual separation of filter and background.

A second explanation considers how the difference between the tristimulus values of the test filter and the predicted constancy filter depends on numerosity. It is obvious that, when the number of randomly drawn colors from a fixed population increases, the mean value of the subset becomes in general more similar to the mean value of the total population. In the most extreme case (that we used as a control condition), the test scene is chromatically identical to the standard scene and thus the proximal match and the constancy match of the filter coincide. However, in the general case, where the predictions from these alternative matching criteria differ, the participants have to find a kind of compromise between both predictions in their filter settings. In this task, a possible strategy could be to combine the two predictions in a constant ratio. This has two implications that are both in line with the observations, namely that the *absolute* deviation of the filter settings from the constancy prediction should increase with the difference between proximal identity and the constancy prediction, and that a *relative* measure like the constancy index should be approximately constant. This assumption could also explain the significantly greater constancy with hazy filters, because the absolute distance between test and standard is here smaller than with a clear filter. This fact is due to the additive constant that reflects the color of the illumination and is itself estimated by the average color of the standard scene.

## Experiment 2: Controlling the scene statistics of the background colors

The chromatic similarity between the subsets used in test and standard scene, and thus also the similarity of the filter colors for identical filter parameters, can be described in terms of the chromatic mean and variance of the corresponding color distributions. Research on color constancy has shown that both the chromatic mean and the chromatic variance can act as cues for the illumination and that the illumination estimation can play a decisive role in material perception.

In the first experiment, these two quantities were confounded naturally with numerosity owing to the random sampling of colors. In our second experiment, we tried to isolate a potential effect of numerosity and, to this end, controlled the selection of the background colors of the subsets in such a way that the correlation between the numerosity of the subset and the deviation of its mean color from the chromatic mean of the parent population was nullified.

As stated in the Introduction, scene constancy for transparent layers was found to be extremely good in Faul and Ekroll (2012). In their scene constancy condition, the reflectances were selected randomly and the illumination was the same as in the standard scene. This practice is similar to the strategy used in our Experiment 1, but a critical difference is that their background colors were afterward corrected so that their mean always exactly corresponded with the illumination color, that is, the spatial mean of the background colors was identical in standard and test. This means that, for identical filter parameters, the filter areas of standard and test have the same average color. It is, thus, not possible to distinguish between a simple proximal match and a constancy match predicted by the filter model. To circumvent this problem in the present investigation, we did not shift subsets with different numbers of colors in color space to match the standard scene in chromaticity, but instead matched pairs of subsets.

### Methods

#### Stimuli

**Figure 11.**
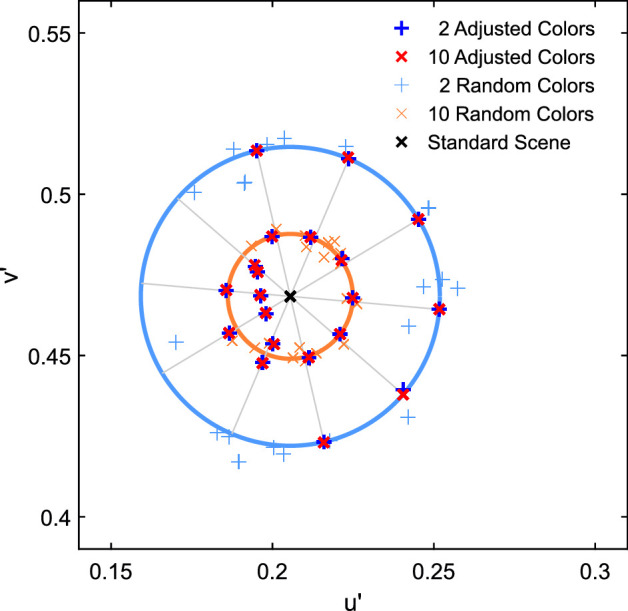
Mean chromaticities of the adjusted subsets used in Experiment 2 for the 2 color subsets (bold blue pluses) and for 10 color subsets (bold red crosses). To illustrate the reasoning behind this selection, also the mean colors of the randomly drawn 2 and 10 color subsets from Experiment 1 are shown. The blue and orange circles indicate the average deviation of the randomly drawn subsets from the mean of the standard scene for 2 and 10 colors, respectively. The aim for the stimuli in Experiment 2 was to adjust the color subsets used in Experiment 1 to match these average deviations in equally distributed directions of the color space (grey spikes). Please note that gamut restrictions of the computer monitor prevented the realization of 10 color subsets with a saturated cyan or bluish spatial mean. We shifted the center of gravity of these subsets toward the achromatic point until all of them were realizable with all filter parameters used.

**Figure 12.**
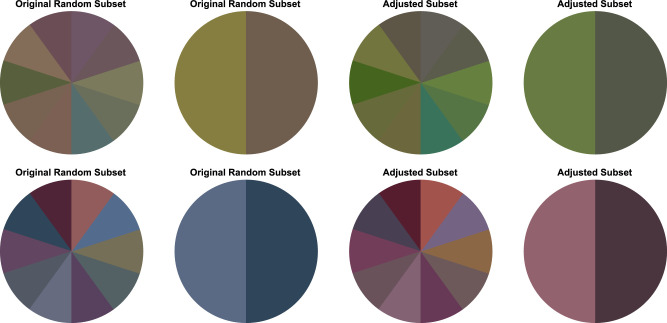
Two examples of 2 and 10 color subsets used in Experiment 1 (left) and Experiment 2 (right). The left two stimuli in each row show the original subsets from the random color selection in Experiment 1 and the two stimuli on the right show the same subsets shifted in u'v'-chromaticity space to share an identical chromatic mean (greenish and reddish).

To control for the chromatic mean of the subsets despite different levels of numerosity, we assembled 20 pairs of subsets with 2 and 10 background colors with pairwise identical mean color. Our goal was to choose 20 subsets that closely match the mean and the variance of the subsets used in Experiment 1 resulting from random selection. Therefore, we first determined the mean deviation of the subsets with 2 versus 10 background colors from the standard scene of Experiment 1 and then shifted the 2 color and the 10 color subsets from Experiment 1 in the u'v'-chromaticity space in such a way that they a) were evenly distributed in all color directions around the standard scene and b) that 10 subsets of each numerosity level exhibited the distant and 10 exhibited the close mean deviation. Figure 11 illustrates the procedure and the result of the stimulus generation for Experiment 2 and Figure 12 shows two examples of shifted subsets.

By this procedure, we accomplished that the mean distance of the subsets from the standard scene was now identical for the 2 color subsets and the 10 color subsets, whereas the chromatic variance and, approximately, also the overall chromatic mean of all 40 subsets were still the same as in Experiment 1. Because the maximal luminance contrast is known to have an impact on the perceived clarity of a filter, we also balanced pairwise for the maximal luminance contrast between both levels of numerosity.

The combination of 10 subsets 2 types of chromatic average (distant vs. close) × two levels of numerosity (2 vs. 10) × three levels of filter hues × two levels of filter clarity resulted in 240 trials. Like in Experiment 1, we conducted five control trials for each of the six investigated filters (30 trials), in which the 162 background colors of the standard scene were also present in the test stimulus (symmetric match) and all scenes containing a blue filter were recorded twice per participant (80 trials). This finally led to 350 trials per participant.

#### Procedure

The procedure was identical to Experiment 1.

#### Participants

Five undergraduate students, different from those who participated in Experiment 1, took part in Experiment 2. All of them were naïve regarding the purpose of the experiment. The experiment was conducted in five or six sessions of about 1.5 to 2.0 hours each. All participants had normal color vision, as assessed by the Ishihara plates (Ishihara, 1967), and reported normal or corrected-to-normal visual acuity. Participation was voluntary and written informed consent was obtained.

### Results

Again, the low overall variance of the settings in the six control conditions speaks for the validity of the filter matches. Because the two repeated measurements for the 80 conditions containing a blue filter were very similar for each observer, their average value was used in the subsequent analyses. After deducting four trials that were marked as erroneous by participants, 1196 data were included in the analysis.

**Figure 13.**
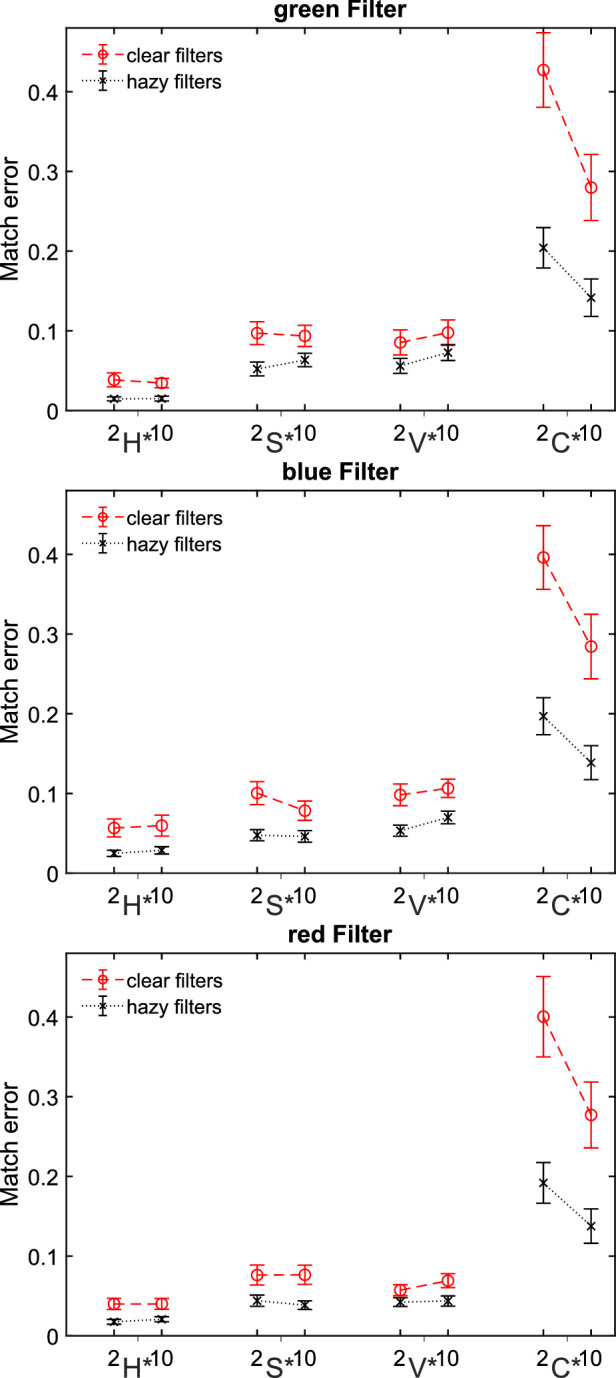
Match errors in Experiment 2. Deviation of the uniform parameter settings hue (H*), saturation (S*), value (V*), and clarity (C*) from the values of the green (top), blue (middle), and red (bottom) filters in the test scene separately plotted for subsets of 2 versus 10 background colors. Each data point is the mean of the absolute error across 100 observations (20 test scenes × 5 participants) minus occasional exclusions. Error bars indicate ± 2 *SEM*.

The observed settings of the uniform filter parameters H*, S*, and V* do not differ systematically between the 2 and the 10 color subsets for any of the three filters examined (Figure 13), whereas the deviations of the clarity parameter setting from the veridical clarity value of the test filter are still more pronounced with 2 background colors than with 10 background colors. Similar to Experiment 1, smaller deviations of all four filter parameters are observed for hazy filters than for clear filters.

**Figure 14.**
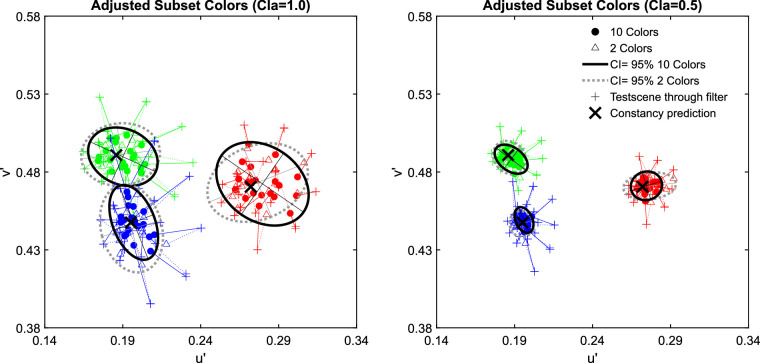
The results of the matching task in Experiment 2 for all three hues of the filter plotted separately for clear filters (left panel, clarity = 1.0) and hazy filters (right panel, clarity = 0.49). The small pluses indicate the positions of the mean color in the filter region for each subset presented as a test scene, that is, the test scene seen through the respective filter and corresponds to the position where a proximal match would lie. The observed match for each subset is connected to the respective proximal match by a dotted line. Each data point corresponds to the chromaticity of the mean filter parameter setting for each subset across all five observers. The matches for the 10 color subsets are marked with filled circles and the matches for the 2 color subsets are indicated by open triangles. The constancy prediction for each filter is indicated with a black cross. The ellipses represent 95% confidence intervals (CI) for the observed matches.

**Figure 15.**
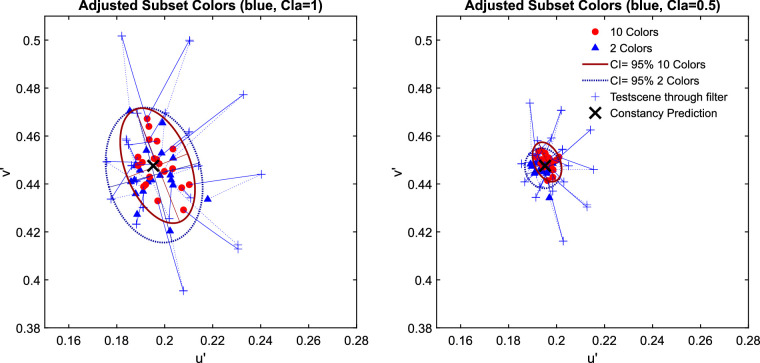
An enlarged view on the data for the blue filter in Figure 14.

**Figure B7.**
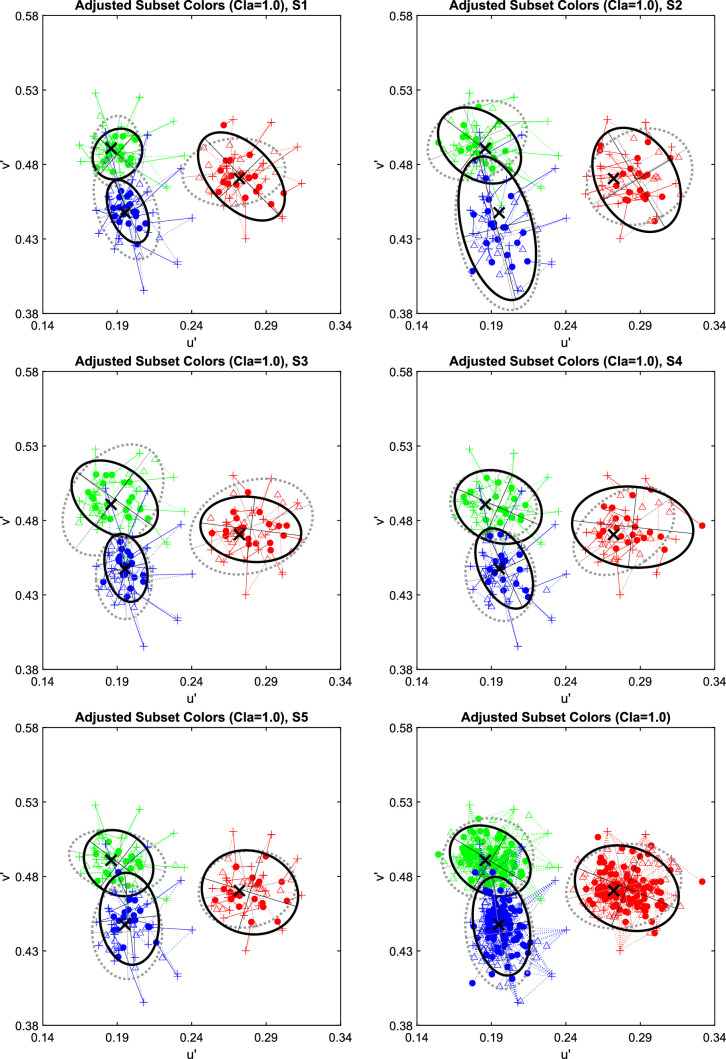
Matching results of the clear filters (clarity = 1.0) in Experiment 2 plotted separately for each participant. The lower right panel contains the data of all observers. The ellipses indicate the 95% CI for the 2 color subsets (dashed line, open triangle) and 10 color subsets (solid line, filled circle). See also the legend of Figure B8.

**Figure B8.**
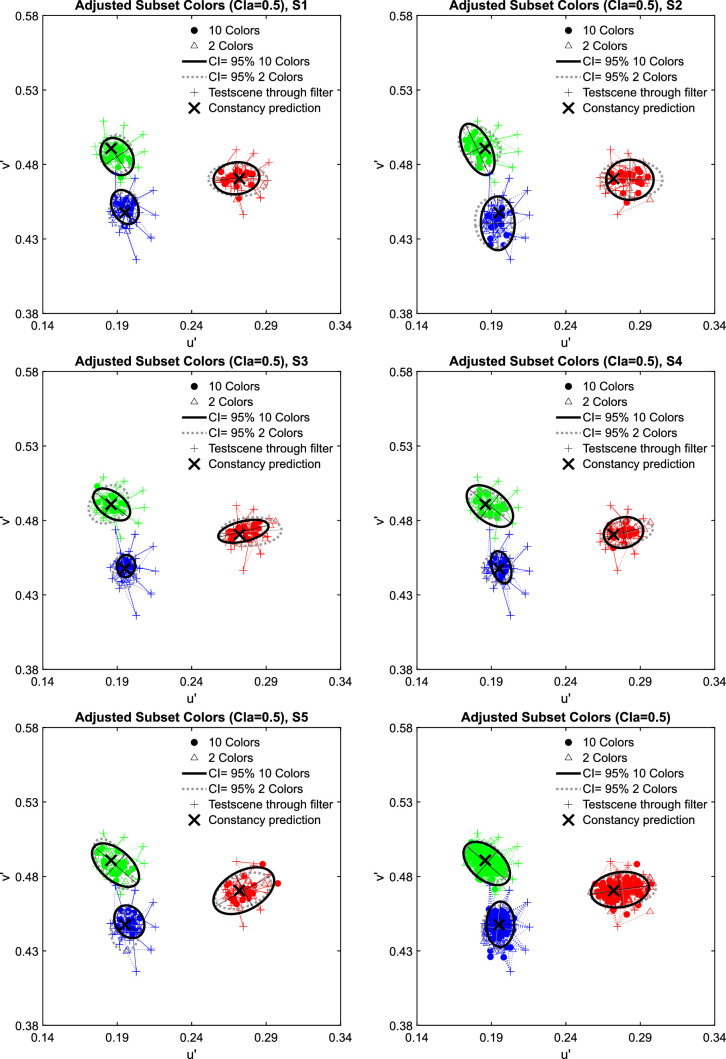
Matching results of the hazy filters (clarity = 0.5) in Experiment 2 plotted separately for each participant (S1–S5). The lower right panel contains the data of all observers.

**Figure B9.**
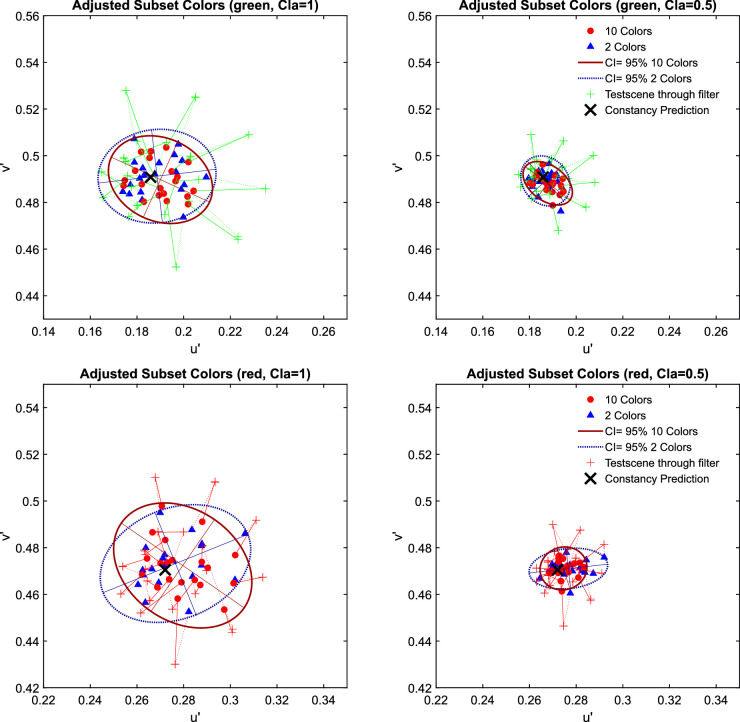
Results for the adjusted subset colors in Experiment 2 for the green and the red filter grouped by the number of subset colors: 2 colors (blue triangle) versus 10 colors (red circle).

The examination of the data in the u'v'-chromaticity space suggests that higher numerosity has no positive effect on transparent layer scene constancy. For all three investigated filter hues the Euclidian deviations of the observed matches from the respective constancy prediction do not systematically differ between the two levels of numerosity (Figure 14). Because the result patterns of all five participants were highly similar, the mean chromaticities were calculated across all participants for each of the 20 test scenes (plots of individual results and the raw data, including scatter ellipses, can be found in Figures B7 and B8 in Appendix B). Figure 15 shows exemplarily for the blue filter the chromaticities of the mean settings across all five observers in more detail (the results for the red and green filter are similar and can be found in Figure B9 in Appendix B).

**Figure 16.**
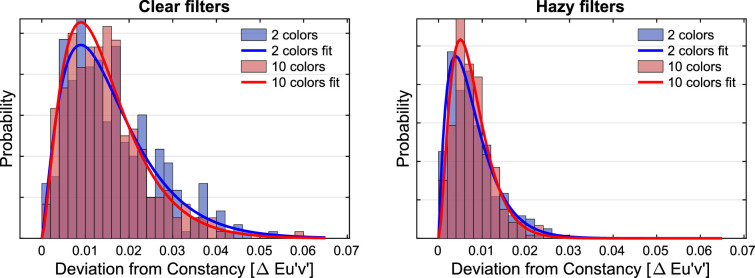
Distributions of the deviations of the matches from the constancy prediction in u'v'-chromaticity space depending on the numerosity of the test scene for clear filters (left) and hazy filters (right). Each of the four distributions refers to 300 measurements (minus occasional exclusions).

For each individual filter setting, the Euclidean distance to the constancy prediction was calculated in the u'v'-chromaticity space. The distributions of these deviations and their dependence on the numerosity of the subset as well as the degree of filter clarity are shown in Figure 16. A simple linear regression suggests that the numerosity is not the relevant predictor for the observed match errors in Experiment 2 with adjusted subset colors (*r* = −0.04, *R*^2^ = 0.001, *df* = 1194, *p* = 0.19).

As in Experiment 1, smaller absolute deviations from the constancy prediction are observed with the hazy filters than with the clear filter, but also in this case there is no longer a difference between the subsets with 2 or 10 colors (Figure 16).

**Figure 17.**
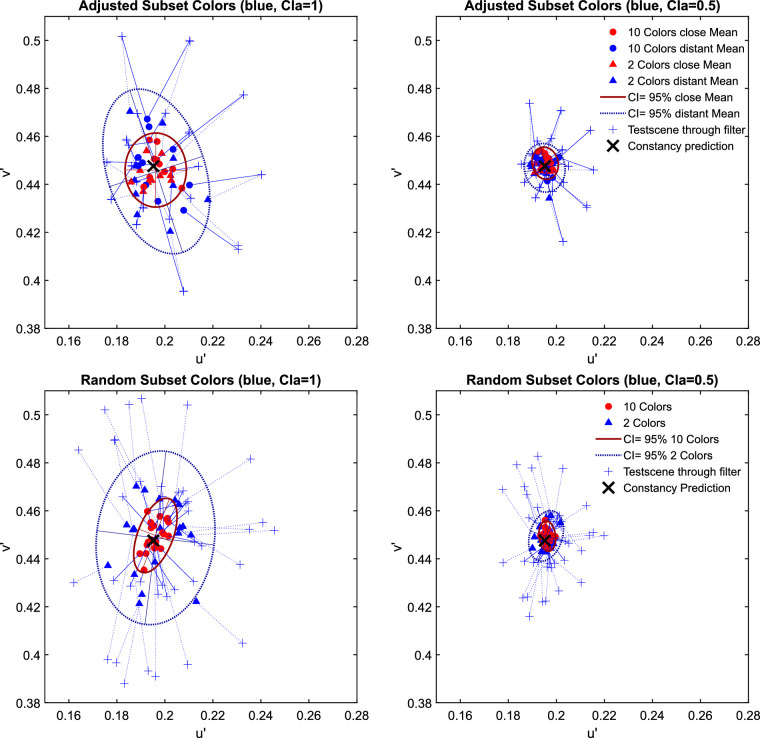
The upper row shows the results of the adjusted subset colors in Experiment 2 exemplarily for the blue filter. The u'v'-chromaticities of the observed filter matches are colored according to the distance between the mean colors of the subset and the standard scene, with red denoting a small and blue a large distance. These results closely resemble those of Experiment 1, which are shown in the lower row for comparison.

**Figure B10.**
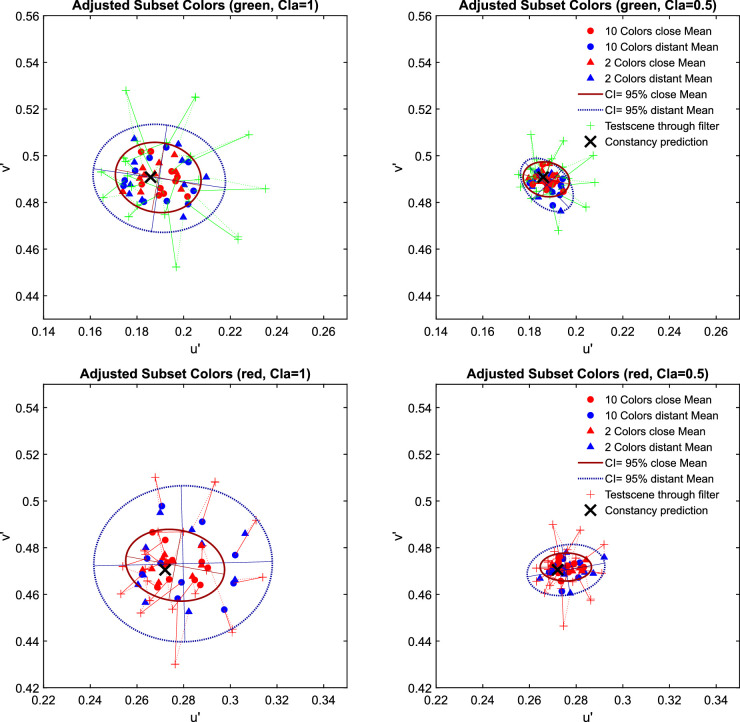
Results for the adjusted subset colors in Experiment 2 for the green (upper row) and the red (lower row) filter. In this figure, the match settings are grouped by the distance of the subsets’ spatial chromatic average to the spatial average of the standard scene, with red denoting a small and blue a large distance. These results closely resemble those of Experiment 1, which are shown in Figure B6.

A completely different picture emerges, however, if the data are grouped by the distance of the mean color of the subset to the mean color of the standard scene and not according to numerosity. Subsets with a large distance between these two colors deviate clearly more from filter constancy, regardless of whether 2 or 10 colors are included in the subset (Figure 17). The resulting picture is remarkably similar to the results from Experiment 1 for each of the tested filter colors. (The results for the red and the green filter grouped by the distance of the subsets’ spatial chromatic average to the spatial average of the standard scene are shown in Appendix B, Figure B10.)

Thus, the responsible factor for the differences in the observed deviations from the constancy filter seems to be the deviation of the chromatic average of the test stimulus from that of the standard scene. In fact, the greater this chromatic deviation is, the greater the deviation of the observed filter matches from the constancy prediction in color space. The result of a simple linear regression of the deviation of the test scene on the match error (*r* = 0.36, *R*^2^ = 0.131, *df* = 1194, *p* < 0.001) shows a similar result to the effect of numerosity in Experiment 1. Again, this effect is more pronounced for the clear filters (*r* = 0.51, *R*^2^ = 0.257, *df* = 597, *p* < 0.001) than for the hazy filters (*r* = 0.27, *R*^2^ = 0.072, *df* = 595, *p* < 0.001).

#### Comparison of randomized and adjusted test subset colors

**Figure 18.**
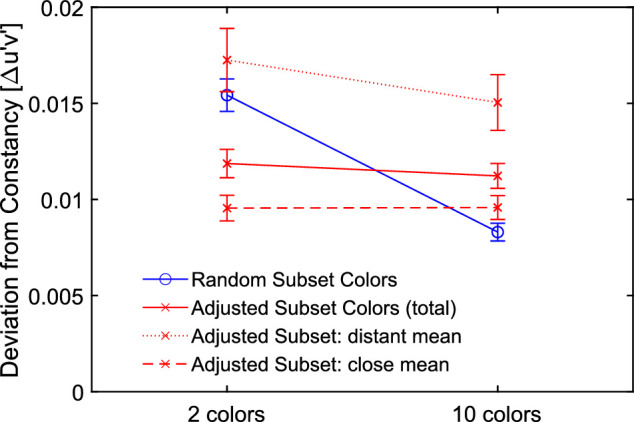
Mean deviations of the observed matches from filter constancy for random color selection in Experiment 1 (blue solid line) and adjusted colors in Experiment 2 (red solid line) for both levels of numerosity. Each data point refers to 600 measurements (minus occasional exclusions) and error bars indicate ± 2 *SEM*. Moreover, the data of Experiment 2 is plotted separately for subsets with a spatial average close (dashed line) and distant (dotted line) to the mean color of the standard scene.

**Figure 19.**
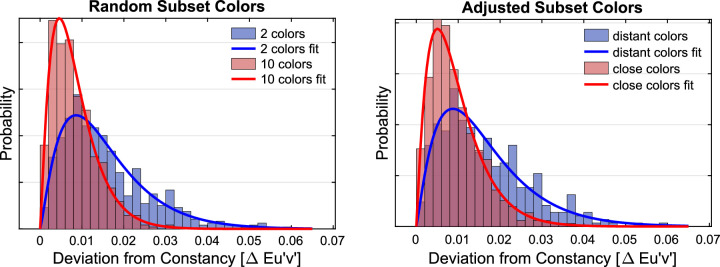
Distributions of the deviations of the matches from the constancy prediction in u'v'-chromaticity space observed in Experiment 1 (left) and Experiment 2 (right). The data for the adjusted subset colors is grouped by distant (blue) and close (red) mean color the subsets from the mean color of the standard scene. The result is quite similar to the effect of numerosity with random subset colors in Experiment 1.

It is evident that the different deviations from filter constancy observed in Experiment 1 with high and low numerosity no longer occur, if the mean background color is kept constant across the two numerosity conditions (Figure 18). However, we found an effect of the distance between the mean colors of the subsets and the standard scene, which is quite similar to the effect of numerosity with random color selection (Figure 19).

**Figure 20.**
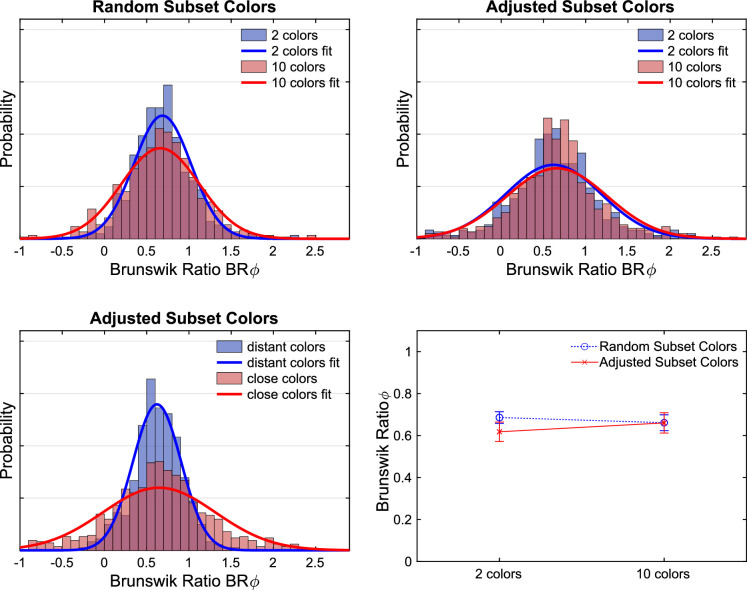
The distribution of the projected Brunswik ratios. The top left panel compares the distributions observed with randomly selected 2 and 10 color subsets. The top right panel shows the corresponding distributions for the adjusted subset colors in Experiment 2. The lower left panel compares the distributions observed for the adjusted subset colors grouped by the means of the subset colors that were either close to or distant from the mean color of the standard scene. The lower right panel shows the mean values observed for 2 and 10 color subsets for both types of subset color selection. Error bars indicate ± 2 *SEM*.

We also calculated the projected Brunswik ratios (BRᵩ) for each filter match, as described in the results section of Experiment 1. We did neither find a difference in the BRᵩ between 2 and 10 color backgrounds nor between the subsets whose mean color is distant or close to the mean color of the standard scene (Figure 20). If this measure actually reflects a compromise between the two match criteria proximal identity and constant filter properties according to the model, then this result would suggest that filter matching is performed according to a fixed ratio between both criteria.

**Figure 21.**
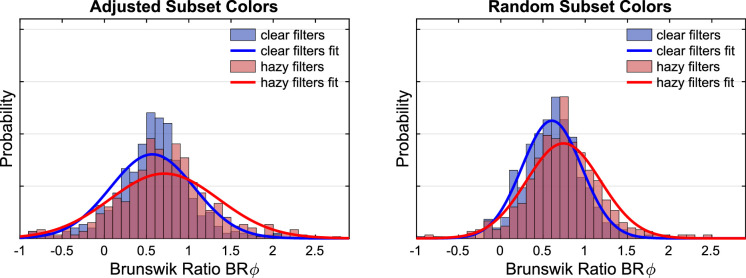
The distribution of the projected Brunswik ratios for clear and hazy filters observed with adjusted subset colors in Experiment 2 (left panel) and with random selection of background colors in Experiment 1 (right panel). It both cases the constancy indices are significantly higher for hazy filters (red) compared with clear filters (blue).

In contrast, there is a clear tendency for a larger BRᵩ in the case of the hazy filters compared with the clear filters for both types of subset color selection (Figure 21). The differences between the BRᵩ for the hazy and clear filters are significant on the 5% level both with adjusted subset colors (*M*_hazy_ = 0.71, *M*_clear_ = 0.58; *t*_238_ = −3.19, *p* = 0.002, two-sided two-samples *t* test) and with random subset colors (*M*_hazy_ = 0.76; *M*_clear_ = 0.61; *t_238_* = −3.98, *p* < 0.001, two-sided two-samples *t* test). Note that the *t* test for each condition of subset color selection is performed on the mean values of the BRᵩ across all five participants for every tested subset and filter condition. This result suggests that the compromise formation changes depending on filter clarity.

**Figure 22.**
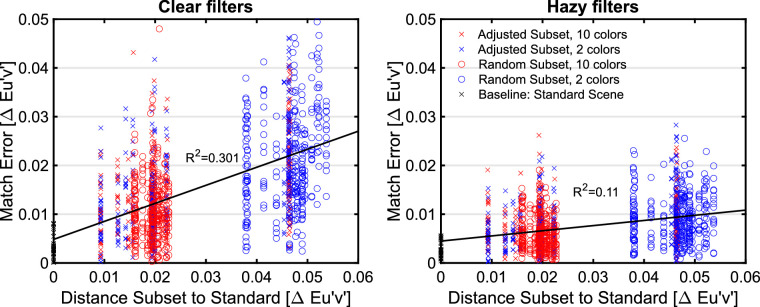
Match error in u'v'-chromaticity space as a function of the distance between the mean colors of subsets and the mean color of the standard scene for all data of both experiments. The results of linear regression indicate that this color distance is a suitable predictor for the deviations from the constancy filter for clear filters (*r* = 0.55, *R*^2^ = 0.301, *df* = 1226, *p* < 0.001), but less important for hazy filters (*r* = 0.33, *R*^2^ = 0.11, *df* = 1223, *p* < 0.001).

The match error in u'v'-chromaticity space that we observed in both experiments is more pronounced with clear filters (Figure 22). The distance of chromatic mean of a test scene from the mean color of the standard scene, however, seems to have less influence in the case of hazy filters.

**Figure 23.**
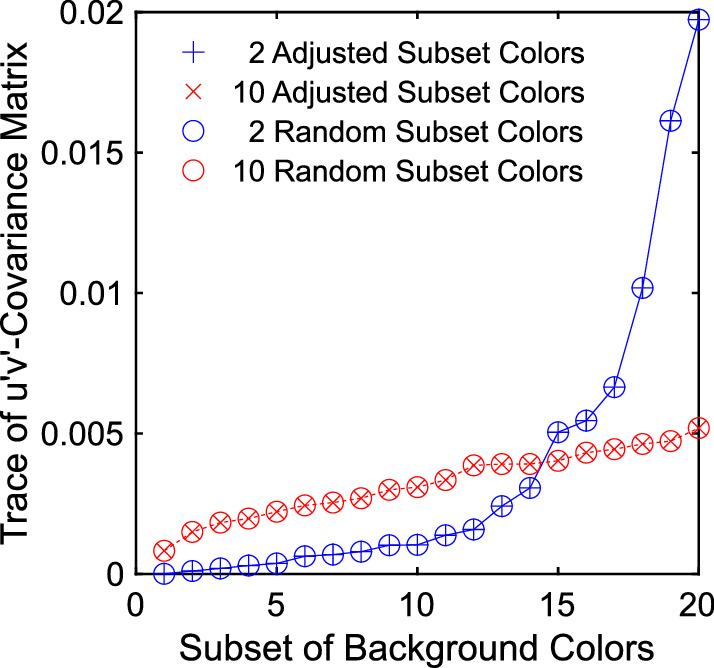
The chromatic variance of each subset is measured by the trace of the u'v'-covariance matrix. Because the subsets used in Experiment 1 were only shifted to different mean colors in Experiment 2, the chromatic variance is pairwise matched and thus constant for both experimental setups.

Because the original randomly drawn subsets of Experiment 1 were simply shifted in u'v'-chromaticity space to control for their mean color in Experiment 2, the chromatic variance was pairwise identical across the two experiments. The variances in the 10 color subsets are rather low and homogeneous, whereas the variances in the 2 color subsets differ more and reach relatively high values (Figure 23). We checked whether the variance of the test scene, which is given by the trace of the u'v'-covariance matrix, could serve as a further predictor of filter constancy. The results of a simple linear regression of the variance on the deviations of the matches from the predicted constancy filter did not support this assumption (*r* = −0.03, *R*^2^ = 0.001, *df* = 2391, *p* = 0.09).

### Discussion

In Experiment 2, the numerosity was again varied in two levels, using subsets with both 2 and 10 colors, but in contrast to Experiment 1 the mean colors in both types of subsets were pairwise identical. Owing to this manipulation, we did no longer observe an enhanced absolute transparent layer constancy for higher levels of numerosity. Instead, the dominant factor determining the degree of transparent layer constancy seems to be the distance of the mean color of the respective subset to the mean color of the standard scene.

Thus, in contrast with a number of results from research on surface color constancy, we did not find an improvement in the degree of transparent layer constancy with an increase in numerosity. The results of Experiment 2 indicate that the crucial factor is not the number of colors itself, that is, the numerosity, but the deviation of the mean color of the subset from the mean color of the standard scene. The adjustment of the background colors nullified the effect of numerosity, which was observed in Experiment 1 when the background colors were randomly selected. This suggests that the mechanisms underlying filter constancy do not “work better” with 10 colors than with 2 colors. The observed results seem compatible with the assumption that a compromise between proximal identity of the filter region and constant filter properties in both scenes is based on a fixed weight ratio. Even though the mechanism does not work better with 10 colors than with 2, the final result may be still better, that is, closer to the constancy prediction, simply because of the fact that expected value of the Euclidean distance between the constancy match and the proximal match is smaller with the 10 color subsets than with 2 color subsets. At least in our experimental setup, the chromatic variance seems not to play an additional role in transparent layer constancy.

For both types of background color selection, the parameter settings for hazy filters were found to be closer to the constancy prediction than those for clear filters. This finding could be attributed to the smaller distance between proximal identity and the constancy prediction in the case of hazy filters. However, this seems not to be the only relevant factor, because higher Brunswik ratios were found for the hazy filters than for the clear filters, which suggests a changed weighting of the two matching criteria. This points to an additional influence on the compromise formation and consequently on the final constancy performance. The question is which properties of the hazy filters lead to the increase in constancy. These filters are characterized by an additive constant, which, according to the filter model, refers to the direct reflection of the illumination at the filter surface. In the case of a homogeneous illumination this lead to a hazy or translucent appearance. The neutral illumination spectrum used for the stimulus generation in our experiments results in an overall brightening of the filter area and this “whiteness” could facilitate the recognition of the filter parameters, especially of filter hue and saturation.

## General discussion

The aim of this work was to investigate the influence of scene articulation on transparent layer constancy. As a first step toward this goal, we used highly reduced abstract scenes and operationalized articulation as the numerosity of background surfaces. The results of Experiment 1 suggest that numerosity does have a crucial effect on transparent layer constancy when the background colors of the test scenes are randomly drawn from the fixed population of a standard scene. We observed greater deviations from a constancy match in all four filter parameters hue, saturation, overall transmittance (value), and clarity for 2 color samples than for 10 color samples. This finding might be explained by the fact that randomly drawn samples of different sizes exhibit different color distributions; in particular, the mean of samples with 10 colors is on average more similar to the standard scene than that of subsets with only 2 colors. However, the effect of numerosity disappears when these natural statistical differences of subsets with different numerosity are artificially nullified as in our second experiment. Then, the mean color of the respective sample appears to be the central predictor for the degree of transparent layer constancy.

A simple effect based on the proximal similarity between test and standard scenes might explain the contrasting influence of numerosity in Experiments 1 and 2. In fact, the more similar the mean background colors in the test and the standard scene, the more similar the filter regions are, both in terms of the constancy prediction according to the filter model and in terms of the proximal similarity, that is, in terms of the mean color in the filter region. Consequently, the two theoretical criteria for a filter match converge, and a constant tradeoff between these two criteria would result in a better match as the test and the standard stimuli become more similar in color. Our finding that the projected Brunswik ratio as a relative measure of constancy did not change with subset sample size supports the hypothesis that the two criteria are weighted by a fixed ratio. The invariant relative constancy index suggests that this fixed ratio between the two perceptual matching criteria is an inherent property of the constancy mechanism.

Thus, in absolute terms, transparent layer constancy increases for a higher number of colors in the test scene, because the proximal similarity between test and standard scene statistically increases with the number of colors drawn. This is in accordance with the analysis of global image statistics by Foster, Amano, and Nascimento (2006), who found the level of illumination-independent color constancy in natural scenes to be slightly improved as the mean hue difference between the two scenes across illuminants decreases.

A second explanation of the effect of numerosity could be based on a more reliable illumination estimation. It has already been pointed out that under natural conditions the mean color of a scene may provide veridical information about the illumination color, even for a small number of background colors (e.g., Faul & Ekroll, 2012), and deviating chromatic means between different scenes seem to provide a dominant cue for an illumination change (e.g., Linnell & Foster, 2002). The enhanced filter constancy for subsets of 10 colors in Experiment 1 is also consistent with the proposal of Mausfeld and Andres (2002) that adding chromatic variance restricts the range of possible illuminants to more neutral lights.

In principle, if a scene is strongly reduced, it is almost impossible to decide whether the present spatial average is due to the illumination or due to the surface reflectances. As the number of randomly drawn reflectances increases, the spatial mean of the scene naturally becomes more and more similar to the illumination color (if the gray-world assumption is true). Thus, when background colors were selected randomly in Experiment 1, subsets of 10 colors always provided a more reliable illumination estimate, which was closer to the veridical achromatic illumination of the standard scene. However, this advantage is lost when, as was done in Experiment 2, the mean color of the subsets with different levels of numerosity is artificially set to the same value.

Our results for transparent layer constancy may also contribute to the field of surface color perception. If the spatial color average of a scene is the decisive cue for the illumination color, then identical spatial color averages of scenes with different numerosity could explain the missing effect of numerosity found in some previous works on color constancy. In fact, several of these works did not control for the mean color of the scenes examined. For example, Arend and Reeves (1986) as well as Arend, Reeves, Schirillo, and Goldstein (1991) argue that the absence of the numerosity effect in their asymmetric color matching experiment may be attributed to the fact that in the low numerosity condition with a bipartite center-surround, Munsell paper N5 was used as the background and therefore corresponded 100% with the illumination color. Because the scenes in their high numerosity condition included 32 color patches that were essentially randomly selected from the Munsell set, it is reasonable to assume that the average background colors of both levels of numerosity did not differ at all or only slightly. Similarly, Radonjić et al. (2015b) could not find an effect for increased numerosity of wallpaper and floor surfaces in rendered scenes when they kept the spatial average between the nonarticulated scenes and the articulated scenes on average constant. Wedge-Roberts et al. (2020) found even worse color constancy for variegated background colors compared with a homogeneous background with a constant spectral reflectance. Although the mean color of the homogeneous gray background corresponded 100% with the illumination color, the global mean color of the variegated scene was not controlled in this experiment.

Furthermore, the results of Lotto and Purves (2000) on simultaneous color contrast demonstrate that the illumination interpretation of a scene seems to be of major relevance. They kept the mean value constant between scenes of low numerosity (one background color) and high numerosity (25 background colors) and then varied the colors in the high numerosity condition such that the resulting color distributions were either consistent with two different illuminations or consistent with a single (achromatic) illumination. In the first case, the simultaneous contrast was enhanced by the higher numerosity, and in the second case, it was significantly attenuated. Such a manipulation of the variance in a high numerosity condition remains to be tested for the filter case.

A second key finding in both experiments is a greater degree of transparent layer constancy for hazy filters than for clear filters. This superiority of the hazy filters was evident not only in the individual filter parameters, but also in an enhanced Brunswik ratio, which would not be expected if a filter match were formed according to a fixed tradeoff between the constancy criterion and the proximal identity criterion. This finding suggests that the weighting of the two criteria changes in the case of hazy filters. These filters are characterized by an additive constant, which corresponds with the direct reflection at the filter surface. Thus, the image of such filters contains the color of the illumination as a separable component, which could serve as a further cue. This result is consistent with enhanced color constancy in the case of specular highlights (Snyder, Doerschner, & Maloney, 2005; Xiao, Hurst, MacIntyre, & Brainard, 2012; Yang & Shevell, 2002). Furthermore, this result supports the hypothesis that a positive effect of numerosity might partly be explained by a more reliable illumination estimate.

However, a limitation of our experimental setup in this regard is that we only used the special case of an achromatic illumination for the stimulus generation. The neutral illumination leads to an overall brightening of the filter area and this “whiteness” may facilitate the recognition of the filter parameter. To verify this assumption, other illuminations need to be investigated to make sure that the distance in color space between the subsets and the standard scene does not coincide with the distance between the subsets and the gray point.

Moreover, although the settings of filter parameters hue, saturation, and value suggest that the colors in the filter area determined by parameter τ of the filter model are more accurately matched for hazy filters, this observation is not unambiguously attributable to a more reliable illumination estimation. This is because for homogeneous illuminations it is not possible to distinguish between the part of the additive constant that is due to τ and the part that is due to the illumination color. To separate the information about the illumination from the information of the filter colors, realistic inhomogeneous illuminations (i.e., illumination maps) would have to be tested in a next step.

Somewhat unexpectedly, we found greater deviations for the clarity parameter with 2 color subsets compared with 10 color subsets. This finding holds even in Experiment 2, despite a pairwise balanced maximum luminance contrast between both levels of numerosity. Variations in luminance contrast are assumed to be the main cause for deviating perceived filter clarity for the same nominal clarity (cf., Faul, 2017). In a simulation with randomly drawn background colors, Faul and Ekroll (2012) showed that the clarity is the poorest estimated parameter of the model when changing the background colors. However, in asymmetric matches with 10 colors in both test and standard, Faul and Ekroll found significantly smaller deviations of the clarity parameter (mean absolute error of <0.08) than we did. For subsets with 10 colors, we found comparable deviations for hazy filters, but significantly larger deviations for clear filters even with 10 colors. It cannot be excluded that these deviations may be due to threshold effects, which depend on the respective starting values of the clarity parameter (in the case of the clarity parameter, numerically larger ranges are perceptually indistinguishable compared with hue values, for example). However, because Faul and Ekroll also used randomized starting values for the clarity parameter and thus potential threshold effects should have played a similar role here, it is not entirely clear how this result may be explained. Unlike Faul and Ekroll, in this experiment we also varied the starting position of the value parameter, so that the filter adjustments might have been more difficult overall.

Finally, for simple abstract 2D stimuli, the question arises whether a specific scene illumination is perceptually represented at all. Available evidence indicates that it is possible to switch between different viewing strategies or perceptual modes such as world versus proximal mode or global versus local mode (cf., e.g., Rock, 1983). Thus, alternation between viewing modes may also contribute to explain the ambiguous findings of the effect of numerosity with comparable stimulus material. Although the chromatic mean may be constant in scenes with low and high numerosity, it is unclear whether it is interpreted in terms of a common illumination. In reduced scenes, the actual viewing mode is unknown, whereas it is rather difficult to adopt a proximal viewing mode in natural scenes of everyday life. An effect of the viewing mode is also supported by a smaller effect of the chosen instructions with naturalistic stimuli compared with simple 2D stimuli (Radonjić & Brainard, 2016).

In this work, we have taken a first step to reveal a taxonomy of cues in natural scenes that are relevant to transparent layer constancy. We started with highly reduced 2D scenes and identified the mean color of such simple scenes as the crucial predictor of transparent layer constancy regardless of the actual number of colors. However, there are indications that other characteristics of natural scenes—beyond numerosity—influence transparent layer constancy, as we found higher degrees of filter constancy for naturalistically rendered scenes compared with 2D stimuli whose colors precisely matched the rendered scenes (Falkenberg & Faul, 2019b). A key distinction between these two types of stimuli was that the rendered scenes featured illumination gradients while the matched 2D color mosaics comprised only uniform color patches. Of course, naturalistic scenes are not necessarily rich in different objects and materials (and thus have a high numerosity), but usually the depicted surfaces are spatially inhomogeneous. Inhomogeneity is caused, for example, by the 3D shape of objects and the different orientations of the object surface to the light source (and to the viewer). The resulting shading is assumed to be a cue for the prevailing illumination and increased lightness constancy was observed in relation to this cue (Boyaci, Doerschner, & Maloney, 2006). Furthermore, there are other sources for inhomogeneous color patches at the image level, such as inhomogeneous reflectance spectra or microsurface irregularities, which are taken into account by newer rendering programs. However, although such “realism cues” may be accounted for in recent rendering programs, they have hardly been investigated so far. The identification of additional cues for transparent layer constancy in natural scenes beyond that of the mean color of a scene is a question yet to be answered.

## Appendix A: The psychophysical filter model and color calculation of the test filter

We refer to a psychophysical filter model formulated in terms of color codes that was proposed in Faul and Ekroll (2002, 2011). The predictions of this model are given by

(A.1) $$P_{i}=\;\tau_{i}\left(A_{i}+\delta I_{i}\right),\;i=\;L,M,S.$$

where *A* denotes the color code of a background region and *P* the code of the same regions viewed through the filter. In this discussion, we always assume that the color codes are cone excitation values, where the index indicates one of *L*, *M*, *S*. The parameters τ, δ, and *I* are related, respectively, to the squared filter transmittance *t*^2^(λ), the direct reflection factor *k*, and the illumination spectrum of the physical model. This interpretation motivates the parameter restrictions 0 ≤ τ_*i*_ ≤ 1 and *d* ≥ 0. The suggested filter model reflects both the subtractive character of the physical model of light transmitting objects, because the parameter τ is directly related to the filter transmittance and the additive character as the parameter δ represents the direct reflection owing to the refractive index. The parameters can be estimated from m ≥ 2 background colors Aj and the corresponding filtered colors Pj that result in regions where the filter (partially) overlaps the background. In Faul and Ekroll (2011), robust estimation techniques were proposed that use the mean and standard deviation (std) of these colors. In a first step, τ is directly estimated by

(A.2) $$\tau_{i}\;=\frac{\mathrm{std}\left(P_{i}\right)}{\mathrm{std}\left(A_{i}\right)},\;i=\;L,M,S.$$

In a second step, δ is integratively estimated via all three color channels by

(A.3) $$\delta =\sum_{i}u_{i}v_{i}/\sum_{i}{v_{i}}^{2},\;i=\;L,M,S.$$

In the last step τ is finally estimated by

(A.4) $${\hat{\tau }}_{i}=\frac{\mathrm{mean}\left(P_{i}\right)}{\mathrm{mean}\left(A_{i}\right)}+\delta \;I_{i},\;i=\;L,M,S,$$

where the index *i* refers to different color channels. As a simple estimate of the illumination parameter *I*, the mean of the background colors *A_i_* can be used. For a model with refractive index 1—as assumed in this article—the parameter δ is zero and the model reduces to

(A.5) $$P_{i}=\;\tau_{i}A_{i},\;i=\;L,M,S,$$

and the unknown parameter τ is proposed to be estimated robustly for *n* ≥ 2 background colors and the corresponding filter colors by

(A.6) $$\tau_{i}\;=\;\mathrm{mean}\left(P_{i}\right)/\mathrm{mean}\left(A_{i}\right)\;,\;i=\;L,M,S.$$

Note that in this case no estimation of the illumination *I* is necessary to calculate τ.

In this way, τ_*Standard*_ can be calculated from the physically generated standard situation. To calculate the filter colors for the test filter, the filter colors *Q_i_* of a constant match are calculated in a next step for the test situation with background colors *B_i_* via *Q*_*i* _ = τ_*Standard*_*B*_*i*_. The filter colors of a proximal match correspond to the filter colors of the standard filter *P_i_*. Thus, in the test situation, the filter parameters τ_*Prox*_ of a proximal match can be calculated via τ_*i*_  =  mean(*P_i_*)/mean(*B_i_*).
